# Inactivity/sleep in two wild free-roaming African elephant matriarchs – Does large body size make elephants the shortest mammalian sleepers?

**DOI:** 10.1371/journal.pone.0171903

**Published:** 2017-03-01

**Authors:** Nadine Gravett, Adhil Bhagwandin, Robert Sutcliffe, Kelly Landen, Michael J. Chase, Oleg I. Lyamin, Jerome M. Siegel, Paul R. Manger

**Affiliations:** 1 School of Anatomical Sciences, Faculty of Health Sciences, University of the Witwatersrand, Johannesburg, South Africa; 2 Elephants Without Borders, Kasane, Botswana; 3 Department of Psychiatry, University of California, Los Angeles, Neurobiology Research 151A3, Sepulveda VAMC, North Hills, California, United States of America; University of Oxford, UNITED KINGDOM

## Abstract

The current study provides details of sleep (or inactivity) in two wild, free-roaming African elephant matriarchs studied in their natural habitat with remote monitoring using an actiwatch subcutaneously implanted in the trunk, a standard elephant collar equipped with a GPS system and gyroscope, and a portable weather station. We found that these two elephants were polyphasic sleepers, had an average daily total sleep time of 2 h, mostly between 02:00 and 06:00, and displayed the shortest daily sleep time of any mammal recorded to date. Moreover, these two elephants exhibited both standing and recumbent sleep, but only exhibited recumbent sleep every third or fourth day, potentially limiting their ability to enter REM sleep on a daily basis. In addition, we observed on five occasions that the elephants went without sleep for up to 46 h and traversed around 30 km in 10 h, possibly due to disturbances such as potential predation or poaching events, or a bull elephant in musth. They exhibited no form of sleep rebound following a night without sleep. Environmental conditions, especially ambient air temperature and relative humidity, analysed as wet-bulb globe temperature, reliably predict sleep onset and offset times. The elephants selected novel sleep sites each night and the amount of activity between sleep periods did not affect the amount of sleep. A number of similarities and differences to studies of elephant sleep in captivity are noted, and specific factors shaping sleep architecture in elephants, on various temporal scales, are discussed.

## Introduction

African elephants (*Loxodonta africana*) are iconic mammals of the continent, and being the largest land mammals have always been of interest in terms of scientific research as an extreme data point for basic physiological functions, such as sleep. Due to their large size, sleep in elephants has been of interest since Benedict and Lee [1] provided the first observations of sleep in circus elephants. This study and several subsequent observational studies (summarized in Table 1), both in captive and wild African and Asian elephants, have developed a generalized picture of sleep in elephants [2–12]. Elephants have been observed to be resting/sleeping while standing [8] with the end of their trunk resting on the ground and usually with their eyes closed, and it has also been observed that elephants can sleep in a recumbent position, during which time REM (rapid eye movement) sleep is presumed to occur, although definitive evidence of rapid eye movements during sleep is still lacking for elephants [8]. The estimates of the daily time spent sleeping by elephants range between 3.10–6.90 hours per day, although the times recorded in the wild setting had lower average total sleep times (3.17–3.36 h/day) [5]. The major sleep episodes for elephants were seen to occur between 22.00 and 07.00, with the time of awakening occurring between 04.30 and 07.00 (Table 1). Thus, elephants appear to be polyphasic, mostly nocturnal sleepers, that appear to wake at around sunrise after approximately 3–6 hours of sleep each night, and that can sleep standing or in recumbency.

**Table 1:** Range of sleep times and other relevant data reported for elephants previously in the literature.

Study	Species	Number and Sex	Setting	Total rest/sleep time (h)	Timing of rest/sleep		Timing of major sleep	Time of awakening	Standing rest/sleep observed?	Recumbent rest/sleep observed?
					Day	Night				
Benedict and Lee, 1938 [1]	Asian and African	Not reported	Captive—Circus	Not reported	Yes, rarely and short duration	Yes	00.00–04.30	04.30–05.00	Yes	Yes
Kurt, 1960 [2]	Asian (10) and African (7)	5 males, 12 females	Captive—Circus	**5.92** h	No	Yes	23.00–07.00	07.00	Yes	Yes
Khüme, 1963 [3]	African	2 males, 1 female	Captive—Zoo	**4.17** h	Not reported	Yes	03.00–05.00	05.00	Yes	Yes
McKay, 1973 [4]	Asian	Both, many animals	Wild–Sri Lanka	Not reported	Yes	Not reported	Not reported	Not reported	Yes	Yes
Wyatt and Eltringham, 1974 [5]	African	6 males, 12 females	Wild—Uganda	**3.17–3.36** h	Yes, short duration	Yes	04.00–07.00	07.00	Yes	Yes
Guy, 1976 [6]	African	27 males, 21 females	Wild—Zimbabwe	Not reported	Yes, around 2.4 h	Not reported	Not reported	Not reported	Yes	Not reported
Adams and Berg, 1980 [7]	African	7 females	Captive—Zoo	Not reported	Yes	Not reported	Not reported	Not reported	Not reported	Yes
Tobler, 1992 [8]	Asian	12 females	Captive–zoo and circus	**3.10–6.90** h	Not reported	Yes	01.00–04.00	06.00–07.00	Yes	Yes
Brockett et al., 1999 [9]	African	3 females	Captive—Zoo	Not reported	Not reported	Yes	01.00–06.00	06.00	Not reported	Yes
Wilson et al., 2006 [10]	African	3 females	Captive—Zoo	Not reported	Not reported	Yes	22.00–02.00	Not reported	Yes	Yes
Horback et al., 2014 [11]	African	7 males, 8 females	Captive—Zoo	Not reported	Yes	Yes	23.00–04.00	05.00–06.00	Yes	Yes
Holdgate et al., 2016 [12]	Asian (28) and African (44)	72 females	Captive—Zoo	African > **2.1** h. Asian > **3.2** h	Yes	Yes	01:00–05:00	Not reported	Yes	Yes

One concern with these studies is that many have been undertaken in the captive setting, with food and water provided and no predation risk, thus not likely reflecting sleep in wild free-roaming elephants. It has been shown [13] that an animal can sleep significantly less in its natural environment than in an artificial environment. That wild elephants appear to sleep less than their captive conspecifics has already been demonstrated [5,8]; however, the observational studies of resting/sleep in wild elephants also suffer from problems such as elephants not being observed at night [4], or conflating rest and sleep [5,6], such that elephants are thought to rest/sleep for up to 2.4 hours during the daylight period [6]. Thus, no unbiased, or remote monitoring, measure of rest/sleep in the wild free-roaming African elephant is currently available.

**Fig 1:**
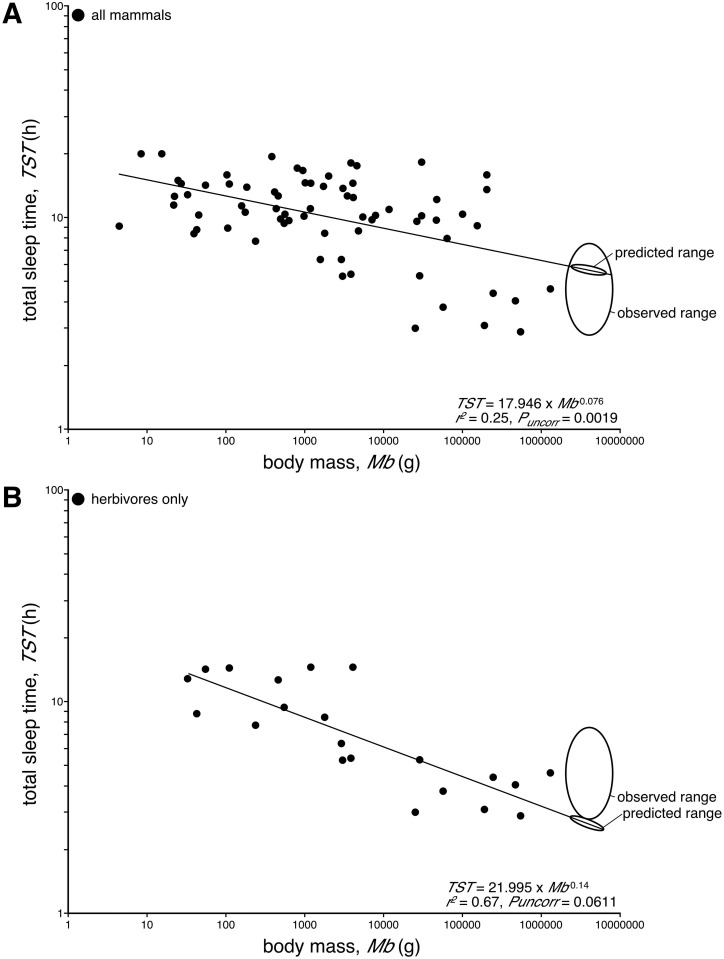
Body mass and total sleep time. Graphs plotting the relationship between body mass (***Mb***, in grams, **g**) and total sleep time (***TST***, in hours, **h**) for all mammals, minus elephant data (**A**) and for herbivores, minus elephant data (**B**). In both cases regressions indicating a lower TST with increased body mass is observed, but this regression, while significant for all mammals, only approaches significance for the herbivores (data used from [14]). Using the derived regression equations, the predicted TST for African elephants has been labelled on each regression line to contrast with observed results from both captive and wild studies (see Table 1).

Can we predict how much a wild free-roaming elephant will sleep? While contentious, there does appear to be a relationship between body mass and total sleep time across mammals, a relationship that is more apparent when only herbivores are analysed [14]. When the elephant data is removed from that used by Siegel [14], a statistically significant relationship between body mass (*M*_*b*_) and total sleep time (*TST*), across all mammals for which data is available, becomes apparent, where *TST* = 17.946 *x Mb* − 0.076 (*r*^*2*^ = 0.25, *P*_*uncorr*_ = 0.0019) (Fig 1A), although only 25% of the variance in *TST* is explained by this relationship. Using this regression equation, African elephants (with adult body masses ranging from 2400–6300 kg) [15] can be predicted to sleep between 5.46–5.87 hours per day. Interestingly, this prediction is within the range reported for *TST* in captive elephants (Table 1, Fig 1A), although it should be noted that the majority of the data used in this regression was obtained from observational studies of captive mammals. When herbivores alone (minus the elephant data) are analysed, the explanatory value of body mass on TST variance increases (higher *r*^*2*^ value), but the regression only approaches significance, such that *TST* = 21.995 *x Mb* − 014 (*r*^*2*^ = 0.67, *P*_*uncorr*_ = 0.0611) (Fig 1B). Using this regression as a predictor, African elephants should sleep between 2.45 to 2.81 hours per day. This prediction is substantially less than that observed in previous studies of elephant sleep in either captivity or the wild (Table 1, Fig 1B).

In addition to this, anatomical studies have highlighted two specializations within the neuronal arousal systems of African elephants indicating that they should be quite short sleepers [16]. It was noted that in the hypothalamus of the African elephant, a medially located parvocellular cluster of orexinergic neurons was present [16]. This parvocellular orexinergic neuronal cluster has only been observed in Cetartiodactyls previously [17,18,19]. Second, a novel medial locus coeruleus nuclear subdivision within the periventricular gray matter of the pons was observed in the African elephant, a feature not observed in any other mammal studied to date [16]. Thus, these two novel nuclei, associated with arousal in other mammals, may contribute to lower sleep times in elephants.

Thus, while there are indications of what sleep should be like, and how long it may last, for wild free-roaming elephants, the picture is not clear. Benedict and Lee [1] made an insightful observation regarding sleep in the elephant that could be translated to studies of sleep in wild elephants, stating: “The degree of trunk movement may well be taken as an index of whether the animal is asleep or awake.” ([1], p. 178]. On this basis we postulated that the use of actigraphy, a commonly used and extensively validated technique for studying sleep and sleep disorders in humans [20], would be particularly useful for quantifying inactivity/sleep in wild free-roaming elephants, creating a remote monitoring system associated with a GPS collar. To this end, we subcutaneously implanted an activity data logger (*Actiwatch Spectrum*, Philips Respironics) in the trunk of each of two wild, free-roaming African elephant matriarchs for a recording period of 35 days and attached a GPS collar. The results of this study reveal a far richer story of elephant rest and sleep than would be predicted from captive studies, and indicate numerous areas for fruitful future research to deepen our understanding of these iconic mammals and their sleep.

## Results

In the current analysis we have considered that inactivity of the trunk for a period of 5 minutes or greater is considered to be a strong sign that the elephant is sleeping. In this sense, we refer to prolonged periods of trunk inactivity as sleep in the current description, with the caveat that trunk inactivity is merely a proxy measurement of sleep in the elephant. Conversely, periods of active trunk movement exclude the possibility of behaviourally defined sleep. Isolated bouts of 5 minutes of trunk inactivity may or may not be accompanied by the full physiological manifestations of sleep, but given the near impossibility of recording even the minimum polysomnographic parameters required to accurately determine physiological sleep in the elephant (in either the captive or wild setting), we feel that this proxy measurement provides an accurate estimate of the maximum amount of sleep of the elephant, although generally actigraphy may overestimate sleep times due to the difficulty in distinguishing quiet wake from sleep using actigraphy alone. Thus, for the remainder of this description we refer to periods of 5 minutes or greater of trunk inactivity as sleep episodes, with these caveats in mind. The raw data analysed and presented in the following sections is provided in Table 2.

**Table 2:** Data used for analysis in the current study. **RD**–recording day; **D**–date as day and month; **SST**–time of sunset; **SRT**–time of sunrise; **MRT–**time of moonrise; **MST**–time of moonset; **MI%**–percentage of moon face illuminated; **SONT**–time of sleep onset; **SOFT**–time of sleep offset; **TST (h)**–total sleep time in hours; **MSEd (h)**–main sleep episode duration in hours; **RSd (min)**–recumbent sleep time in minutes; **WBGTmin (°)**–minimum wet bulb globe temperature in degrees; **WBGTminT**–time of minimum wet bulb globe temperature; **WBGT SON (°)**–wet bulb globe temperature at sleep onset; **WBGT SOF (°)**–wet bulb globe temperature at sleep offset; **SLD MSEs (km)**–straight line distance between main sleep episode sites in kilometres; **D MSEs (km)**–total distance traversed between main sleep episodes sites in kilometres; **D M1/M2 MSEs (km)**–distance between main sleep episode sites of matriarch 1 and matriarch 2 in kilometres; **TAC MSE**–sum of trunk activity counts between main sleep episodes; **M1** –matriarch 1; **M2** –matriarch 2; **X**–days without data as the elephants did not sleep on these days.

RD	D	SST	SRT	MRT	MST	MI%	SONT		SOFT		TST (h)		MSEd (h)		RSd (min)		WBGT min (°)	WBGT minT	WBGT SON (°)		WBGT SOF (°)		SLD MSEs (km)		D MSEs (km)		D M1/M2 MSEs (km)	TAC MSE	
							M1	M2	M1	M2	M1	M2	M1	M2	M1	M2			M1	M2	M1	M2	M1	M2	M1	M2		M1	M2
1	14 Apr	18:11	6:32	16:52	5:31	99.8	3:30	1:05	6:10	4:25	3.3	2.1	0.92	0.33	0	37	18.42	6:00	22.14	23.33	18.53	21.45	2.70	17.64	7.07	22.1	10.32	X	X
2	15 Apr	18:11	6:32	17:31	6:27	100.0	1:35	2:40	6:20	4:55	4.2	1.2	1.58	0.42	69	0	17.7	6:40	21.14	19.7	17.65	19.13	2.20	9	6.84	16.35	3.98	206259	331423
3	16 Apr	18:10	6:32	18:12	7:24	99.6	3:40	0:15	6:00	4:45	2.2	1.9	0.83	0.75	57	0	16.61	7:00	17.94	19.3	17.43	17.4	2.77	1.36	9.04	11.65	0.18	243346	305782
4	17 Apr	18:10	6:32	18:57	8:22	96.8	X	X	X	X	X	X	X	X	0	0	17.59	2:50	X	X	X	X	X	X	X	X	X	X	X
5	18 Apr	18:09	6:33	19:46	9:22	91.6	1:05	1:40	3:45	3:55	2.4	2.0	1.75	0.83	0	0	17.92	5:20	20.12	19.45	18.57	18.4	29.26	29.07	54.92	51.49	1.30	514192	685219
6	19 Apr	18:09	6:33	20:38	10:20	84.0	19:00	3:10	5:10	5:00	3.6	1.0	2.17	0.58	0	0	15.23	4:40	24.73	15.85	15.67	15.53	3.66	4.23	5.1	14.1	2.53	179234	409785
7	20 Apr	18:08	6:33	21:35	11:17	74.5	2:55	3:05	6:00	5:55	2.9	2.8	1.42	1.33	0	28	14.64	5:30	17.75	17.66	15.83	15.83	4.02	1.61	13	10.53	4.16	396007	401057
8	21 Apr	18:08	6:33	22:35	12:10	63.6	1:45	1:50	4:10	4:45	2.3	2.1	1.92	0.67	0	66	14.38	6:50	17.69	17.69	16.06	15.25	2.23	2.07	7.81	12.2	4.72	276659	360353
9	22 Apr	18:07	6:34	23:36	13:01	51.8	2:25	23:50	4:55	5:55	2.2	3.3	1.75	1.25	122	0	16.02	1:40	17.11	19.69	18.31	16.82	3.21	0.98	19.21	10.85	7.71	297731	402186
10	23 Apr	18:07	6:34	0:38	13:48	39.9	1:05	1:25	5:55	5:35	2.8	1.3	1.58	0.50	104	0	15.5	6:40	18.42	18.18	15.91	15.96	0.84	4.17	13.08	15.36	9.30	291464	368946
11	24 Apr	18:06	6:34	1:39	14:32	28.6	2:15	4:50	3:15	5:30	0.8	0.5	0.42	0.17	0	0	18.66	3:30	20.25	20.48	18.67	19.84	11.16	2.95	15.67	16.39	1.74	292220	497660
12	25 Apr	18:06	6:35	2:40	15:15	18.5	23:05	1:45	2:05	5:10	2.7	2.2	1.75	0.75	0	43	18.95	6:40	22.35	21.93	21.94	21.38	10.50	8.97	20.3	18.39	0.15	293754	393526
13	26 Apr	18:05	6:35	3:40	15:57	10.3	0:05	2:00	5:55	5:25	2.8	0.4	1.33	0.17	46	0	20.98	4:20	23.21	22.16	21.97	21.78	13.91	14	22.02	20.76	0.21	324370	401567
14	27 Apr	18:04	6:35	4:40	16:39	4.3	1:15	2:00	6:00	4:40	4.0	2.3	1.25	0.50	0	29	20.76	4:10	22.23	22.4	21.62	21.16	7.72	8.36	18.19	15.76	0.63	230105	408509
15	28 Apr	18:04	6:35	5:39	17:22	0.9	4:00	2:30	6:05	5:45	1.9	2.2	1.75	0.33	18	0	14.11	5:00	15.12	16.86	14.47	14.39	8.26	7.44	16.69	16.33	0.25	283932	353663
16	29 Apr	18:03	6:36	6:37	18:07	0.0	23:15	1:10	5:55	4:50	5.0	2.8	1.83	1.25	27	73	14.96	3:20	18.6	16.3	15.08	15.45	4.52	3.01	9.92	9.87	6.87	225604	323799
17	30 Apr	18:03	6:36	7:35	18:54	1.7	2:30	0:25	6:15	4:10	4.5	3.0	3.75	1.58	93	47	12.95	6:40	14.02	14.71	13.48	14.87	4.88	0.54	8.42	10.49	10.12	237400	334396
18	1 May	18:02	6:36	8:30	19:42	5.6	23:00	23:45	5:40	4:50	2.7	2.5	1.33	1.17	0	56	11.92	6:30	16.95	16.05	12.45	12.95	3.21	1.59	8.07	7.71	10.60	200556	353958
19	2 May	18:02	6:37	9:23	20:32	11.3	0:30	1:00	6:10	5:45	4.0	3.1	1.58	1.75	0	0	13.32	6:40	16.28	16.08	13.72	13.82	3.71	1.73	11.95	10.63	12.52	233373	356489
20	3 May	18:01	6:37	10:12	21:23	18.6	4:05	23:05	5:55	5:20	1.6	3.3	0.33	1.08	0	0	13.26	5:30	13.97	16.62	14.46	13.62	2.14	0.99	11.98	11.81	10.87	276196	306004
21	4 May	18:01	6:37	10:59	22:13	27.0	2:30	2:45	6:00	4:50	2.9	1.5	1.50	0.92	0	0	11.18	6:30	15.95	16.26	11.46	12.23	0.97	1.43	11.01	7.88	12.63	239936	344733
22	5 May	18:00	6:38	11:41	23:04	36.1	1:10	X	6:00	X	3.2	X	1.42	X	0	0	11.59	6:30	15.7	X	11.81	X	1.23	X	10.96	X	X	237326	X
23	6 May	18:00	6:38	12:21	23:55	45.7	1:35	21:20	4:45	5:10	2.9	3.6	1.33	2.33	142	26	14.58	7:00	15.71	19.98	14.8	14.82	5.43	26.86	9.88	47.88	34.05	214613	673390
24	7 May	17:59	6:38	12:59	0:45	55.5	2:50	2:00	5:35	5:45	2.5	1.4	1.08	0.42	0	0	16.55	6:50	19.56	19.91	17.4	17.28	4.41	10.32	9.92	20.14	25.12	253618	396942
25	8 May	17:59	6:39	13:36	1:36	65.2	X	2:00	X	4:05	X	1.3	X	0.92	0	0	15.53	6:50	X	18.43	X	15.64	X	0.73	X	27.28	X	X	288319
26	9 May	17:58	6:39	14:12	2:27	74.4	22:15	2:55	3:40	4:40	2.7	1.3	1.83	0.42	0	0	13.88	6:00	20.2	15.89	15.83	15.37	40.21	5.37	66.41	16.73	17.70	451563	370766
27	10 May	17:57	6:39	14:48	3:20	82.8	3:35	2:00	5:00	6:20	1.2	1.6	0.83	0.42	0	0	13.27	6:50	14.85	15.14	13.61	13.3	2.62	0.65	9.09	11	16.76	353840	330753
28	11 May	17:57	6:40	15:26	4:14	90.0	3:30	3:15	5:35	5:15	2.2	1.8	2.08	0.75	0	0	12.89	6:30	14.09	14.08	13.46	13.45	9.34	3.85	14.77	9.06	4.23	318708	338533
29	12 May	17:56	6:40	16:06	5:11	95.5	4:05	3:20	4:55	3:35	0.8	0.3	0.67	0.25	0	0	15.05	7:00	17.77	17.71	16.22	17.89	8.19	1.7	19.05	28.3	4.34	276893	341444
30	13 May	17:56	6:40	16:49	6:09	99.0	0:50	2:25	4:30	3:40	1.0	1.3	0.50	0.50	0	0	11.84	6:30	13.9	13.65	13.48	13.62	15.30	5.49	24.92	13.92	16.96	305825	359688
31	14 May	17:55	6:41	17:35	7:10	100.0	X	2:10	X	4:35	X	1.8	X	1.33	0	0	8.34	6:40	X	11.71	X	8.95	X	5.1	X	8.83	X	X	312158
32	15 May	17:55	6:41	18:29	8:10	99.9	22:50	0:00	5:45	3:45	1.3	3.4	0.83	1.08	0	48	9.22	6:30	13.88	16.27	9.55	11.04	9.52	11.83	31.95	19.8	4.90	634010	289410
33	16 May	17:54	6:41	19:26	9:09	98.1	1:15	2:50	3:45	4:55	1.8	1.8	0.42	1.42	41	0	10.61	5:50	12.59	12.06	11.48	11.06	5.16	13.35	12.17	19.2	16.44	354017	363174
34	17 May	17:54	6:42	20:26	10:06	93.5	1:35	3:15	3:05	3:50	0.4	0.6	0.08	0.33	0	0	11.16	6:50	13.93	12.82	13.2	12.07	1.51	2.06	6.77	14.02	15.74	319731	329249
35	18 May	17:53	6:42	21:29	11:03	86.3	21:40	2:20	3:50	5:50	0.6	1.3	0.17	0.50	0	0	12.52	4:00	18.08	14.37	12.62	12.97	6.62	18.4	7.78	24.97	8.38	268639	364092

### Total sleep times, polyphasic sleep and timing of sleep

Total daily sleep time (TST) in Matriarch 1 averaged 2.3 h (s.d. = 1.3 h, range = 0–5 h), while for Matriarch 2 the TST averaged 1.8 h (s.d. = 1.0 h, range = 0–3.6 h). If the data for both elephants is combined, the average TST is 2.0 h (s.d. = 1.2 h). Despite this, during the recording period there were four days in which the elephants recorded from did not sleep, or were inactive for less than 10 minutes (see below for full details), and this slightly increases these average times and slightly reduces the standard deviation. Thus, when these days of minimal to no sleep are eliminated from the analysis, TST in Matriarch 1 averaged 2.5 h (s.d. = 1.2 h, range = 0.4–5 h), for Matriarch 2 TST averaged 1.9 h (s.d. = 0.9 h, range = 0.3–3.6 h), while for both elephants combined, the average TST was 2.2 h (s.d. = 1.1 h).

**Fig 2:**
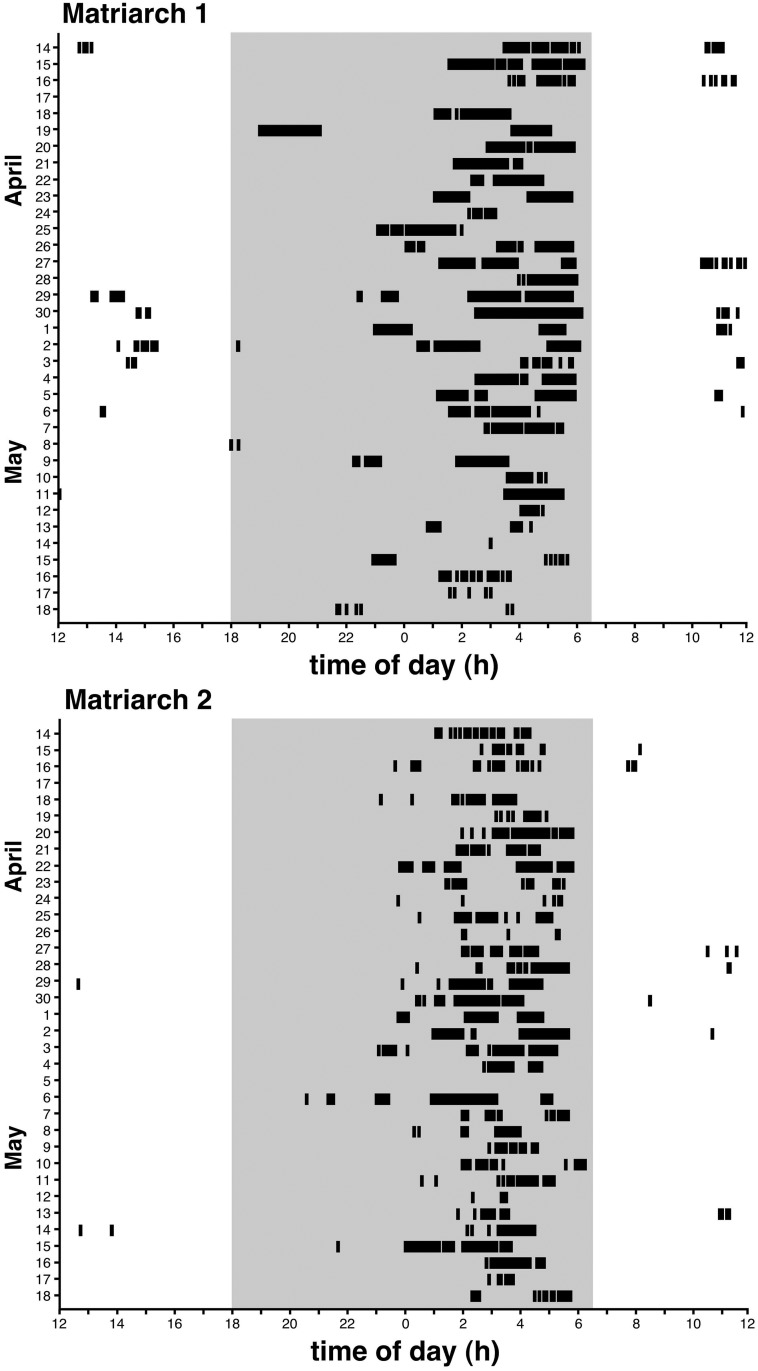
Elephant inactigrams. Inactigrams for each elephant during the entire recording period, from April 14 to May 18. Each solid mark indicates a period of 5 minutes or more when the trunk of each elephant was completely immobile, the elephant presumably being asleep. The grey regions represent the period between sunset and sunrise. Note that the vast majority of sleep occurred during the hours leading up to sunrise, but occasional sleep episodes could occur at different times throughout the 24-h period.

**Fig 3:**
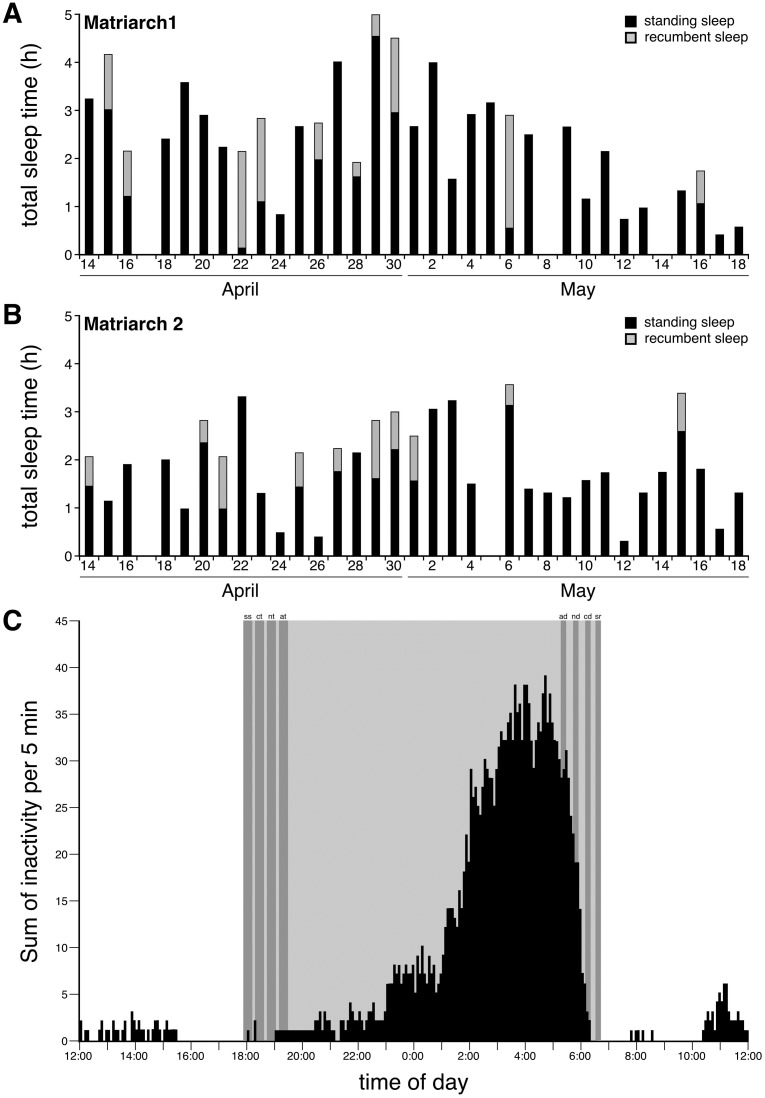
Sleep times, episodes and timing in the elephant. **A** and **B**. Bar graphs representing total sleep time on each day through the 35-day recording period for each elephant, **A**–Matriarch 1, **B**–Matriarch 2. Note that on certain days no sleep was observed. The bars also represent the amount of time spent in standing sleep (**black**) and in recumbent sleep (**grey**), although recumbent sleep did not occur on each day. **C**. Graph illustrating the average count of inactivity/sleep episodes for any given 5 min period scored over the 35 day recording period and combining the data from both elephants. Note the clearly nocturnal pattern of inactivity, with little inactivity occurring during the daytime. The vast majority of sleep episodes occurred in the early morning during the hours of 02:00 and 06:00 (see also Fig 2). The grey region represents the period between sunset (**ss**) and sunrise (**sr**). astronomical dawn–**ad**, astronomical twilight–**at**, civil dawn–**cd**, civil twilight–**ct**, hour–**h**, nautical dawn–**nd**, nautical twilight–**nt**.

Sleep in the two elephants did not occur in a single episode, rather, each day where sleep occurred (see below for exceptions) it consisted of multiple episodes, making the elephant a polyphasic sleeper (Figs 2, 3A and 3B). Matriarch 1 had an average of 4 sleep episodes per day (s.d. = 3; range = 0–11), Matriarch 2 had an average of 5 sleep episodes per day (s.d. = 2; range = 0–11), and combined the average number of daily sleep episodes were 5 (s.d. = 3). In each day there was one main episode of sleep (the longest episode of daily sleep), with a number of shorter episodes of sleep occurring mostly prior to the main sleep episode, but also on occasion occurring after the main sleep episode (Fig 2). The main sleep episode (excluding the days in which the elephants showed no signs of sleep, see below) in Matriarch 1 averaged 1.3 h (s.d. = 0.7 h; range = 0.1–1.9 h), in Matriarch 2 the average duration of the main sleep episode was 0.8 h (s.d. = 0.5 h; range = 0.17–1.8 h), while combined the main sleep episode averaged 1.1 h (s.d. = 0.7 h). For the remaining non-main episodes of sleep, in Matriarch 1 the average duration of these episodes was 22.3 min (s.d. = 18.8 min; range = 5.0–85.0 min), for Matriarch 2 the average duration was 14.8 min (s.d. = 8.6 min; range = 5.0–40.0 min), while combined the average duration of these non-main sleep episodes was 18.6 min (s.d. = 15.0 min).

The timing of sleep in the elephant was clearly nocturnal (Fig 2), with 94.6% of all 5 min epochs of sleep occurring between sunset and sunrise (Fig 3C). The vast majority of this nocturnal sleep occurred between 01:00 and 06:00, although sleep episodes could be observed earlier in the night. While there appears to be some episodes during daylight that could be classified as “napping”, these were limited and did not occur on a daily basis, and appeared to occur mostly between 10:30 and 15:30 (Fig 3).

### Recumbency during sleep

In order to determine whether the wild free-roaming elephants ever slept in a recumbent position, as seen in studies of captive elephants (see Table 1), we analysed data from a gyroscope placed within the collar and correlated it with the episodes of trunk inactivity scored as sleep. For each elephant we found evidence of recumbent sleep on only 10 of the 35 days of recording, although these were on different days for each elephant, and the remaining 25 days for each elephant showed no recumbent sleep (Fig 3A and 3B). For Matriarch 1, the total amount of time spent in recumbent sleep over the 35-day recording period was 719 min (11 h 58.8 min), while Matriarch 2 spent 453 min (7 h 33 min) in recumbent sleep. As an overall average, Matriarch 1 spent 20.54 min (s.d. = 39.33 min; range = 0 to 142 min) per day in recumbent sleep, while Matriarch 2 spent 12.94 min (s.d. = 22.35 min; range = 0 to 73 min) in recumbent sleep; however, as mentioned each elephant only exhibited recumbent sleep on 10 of the 35 days of recording, with Matriarch 1 having 12 episodes of recumbent sleep and Matriarch 2 having 14 episodes. For Matriarch 1 only one recumbent sleep episode was recorded on 8 of the 10 days when recumbent sleep occurred (with 2 episodes per day on the remaining 2 days), while for Matriarch 2 only one recumbent sleep episode was recorded on 6 of the 10 days when recumbent sleep occurred (with 2 episodes per day on the remaining 4 days). The episodes of recumbent sleep had an average duration of 65.35 min (s.d. = 42.55 min; range 18 to 142 min) for Matriarch 1, while recumbent sleep episodes had an average duration of 37.75 min (s.d. = 17.60 min; range = 4 to 73 min) for Matriarch 2. As a proportion of TST, Matriarch 1 spent 15.17% of TST in a recumbent position, while Matriarch 2 spent 12.00% of TST in a recumbent position. Matriarch 1 was a “left-trunker”, while Matriarch 2 was a “right-trunker” (see methods section). Of the time spent in recumbent sleep, Matriarch 1 spent 48.4% lying in her left side, and 51.6% lying on her right side, while Matriarch 2 spent 71.3% lying on her left side and 28.7% lying on her right side, indicating no relationship between trunk side preference and recumbent sleep side preference.

### Timing of sleep onset and offset (Awakening)

**Fig 4:**
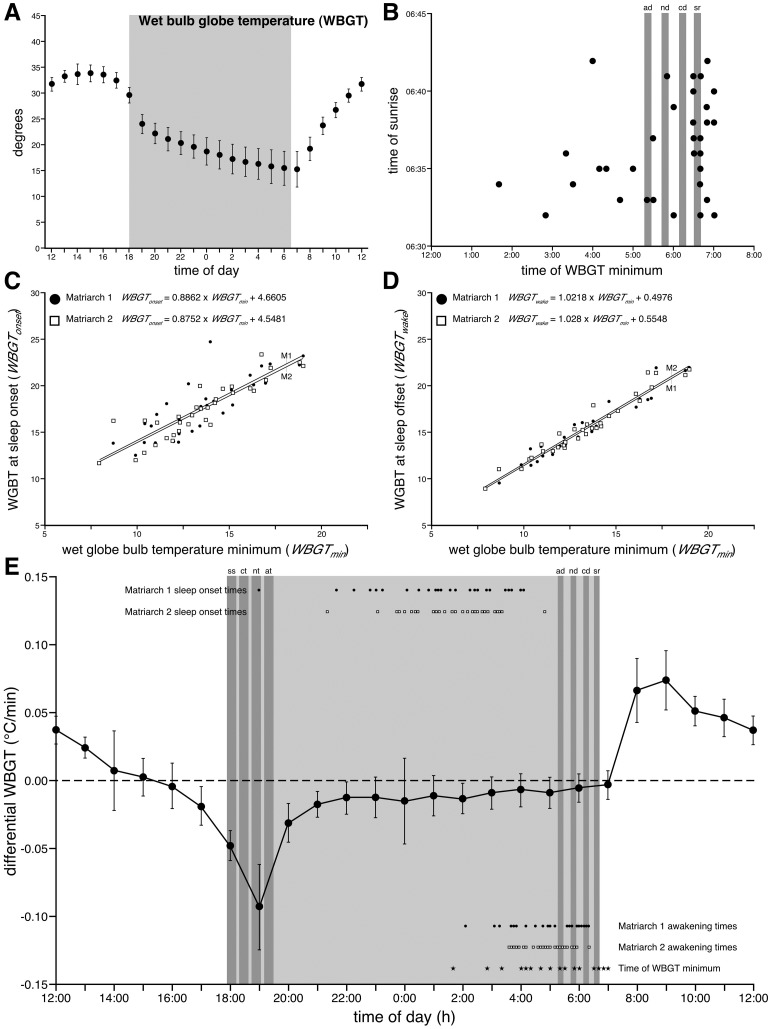
Wet globe bulb temperature and sleep in elephants. Graphs showing the relationships between wet globe bulb temperatures (**WBGT**) and the timing of sleep onset and offset/awakening in the two elephants studied. **A**. This graph shows the hourly averages (with the bars representing 1 standard deviation) for WBGT during the 35-day recording period. The grey region represents the period between sunset and sunrise. **B**. This graph depicts the relationship between the timing of the minimum WBGT and the various dawn times (dark grey bars), including sunrise (**sr**), civil dawn (**cd**), nautical dawn (**nd**) and astronomical dawn (**ad**). **C**. Graph representing the statistically significant linear relationships between minimum WBGT (***WBGT***_***min***_) and WGBT at sleep onset (***WBGT***_***onset***_) for both elephants (Matriarch 1 –**M1**; Matriarch 2 –**M2**). **D**. Graph representing the statistically significant linear relationships between minimum WBGT (***WBGT***_***min***_) and WGBT at sleep offset/awakening (***WBGT***_***wake***_) for both elephants. **E**. Graph showing the relationships between the hourly changes in WBGT averaged over the 35 day recording period, the daily sleep onset and awakening times for both elephants, the timing of the daily minimum of WBGT, and the specific sunset (including sunset–**ss**, civil twilight–**ct**, nautical twilight–**nt**, and astronomical twilight–**at**) and sunrise times. Note that sleep onset, time of sleep and sleep offset/awakening all occurred during the period when the WBGT was slowly dropping, and do not coincide with the rapid periods of cooling (around sunset) or warming (after sunrise).

The time of sunset was 18:11 at the start of the 35-day recording period, and was 17:53 at the end of the recording period. Sleep onset was defined as the beginning of the first consolidated period of sleep, greater than 10 minutes, following sunset. The timing of sleep onset varied during the recording period, but for Matriarch 1 the average time of sleep onset was 01:20 (s.d. = 127 min; range 19:00 to 04:05), for Matriarch 2 the average time of sleep onset was 01:45 (s.d. = 88 min; range 21:20 to 04:50), while combined the average time of sleep onset was 01:33 (s.d. = 109 min) (Fig 4E). Matriarch 1 went to sleep on average 7 h 18 min (s.d. = 127 min; range = 51 to 609 min) after sunset, Matriarch 2 went to sleep on average 7 h 43 min (s.d. = 89 min; range = 200–664 min) after sunset, while combined the average time of sleep onset after sunset was 7 h 31 min (s.d. = 109 min). Thus, the two elephants studied always went to sleep a substantial time after sunset, but the timing of sleep onset was quite variable (Fig 4E). The timing of sleep onset was also substantially after civil twilight (which occurred 22 min after sunset), nautical twilight (which occurred 47 min after sunset) and astronomical twilight (which occurred 73 min after sunset). No clear correlations indicate that sunset, of any sort, played a significant role in sleep onset.

Due to the lack of a relationship between sleep onset and sunset times we examined the relationship of minimum wet bulb globe temperature (WBGT) to the timing of sleep onset. The timing of minimum WBGT varied throughout the recording period, but the minimum WBGT was recorded on average at 05:46 (s.d. = 82.8 min; range = 01:40 to 07:00). Minimum WBGT occurred on average 50.5 min (s.d. = 81.9 min; range = 196 min prior to 28 min after sunrise) prior to sunrise, and while minimum WBGT often occurred at a regular time after sunrise, there was a great deal of variability of timing of minimum WBGT, providing no consistent relationship with the timing of sunrise (Fig 4B). Sleep onset generally occurred substantially earlier than the minimum WBGT (Fig 4E). Sleep onset for Matriarch 1 occurred prior to minimum WBGT for 31 out of 32 sleep onset events (96.9%), while for Matriarch 2 sleep onset occurred prior to minimum WBGT for 32 out of 33 sleep onset events (97.0%) (see below for days without sleep). Sleep onset for Matriarch 1 was, on average, 268.13 min (4 h 28.13 min; s.d. = 135.0 min; range = 580 min prior to 45 min after) prior to minimum WBGT, while sleep onset for Matriarch 2 was, on average, 258.64 min (4 h 18.64 min; s.d. = 131.6 min; range = 580 min prior to 80 min after) prior to minimum WBGT (Fig 4A and 4E). Thus, sleep onset in the two elephants studied generally occurred prior to the time of minimum WBGT (Matriarch 1: p = 1.85 x 10^−12^; Matriarch 2: p = 1.07 x 10−^12^; paired T-test), and while this is a statistically significant result, the variance is high (Fig 4E).

The WBGT at the time of sleep onset was, on average, 2.99 degrees higher than the minimum WBGT for Matriarch 1 (s.d. = 1.87 degrees; range = 0.71 to 9.50 degrees), while for Matriarch 2 it was on average 2.72 degrees (s.d. = 1.39 degrees; range = 0.62 to 7.05 degrees) higher than the minimum WBGT. Interestingly, statistically significant linear correlations existed between minimum WBGT (*WBGT*_*min*_) and WGBT at sleep onset (*WBGT*_*onset*_) for both elephants (Fig 4C), such that for Matriarch 1:

$$WBGT_{onset}=0.8862\;x\;WGBT_{min}+4.6605\;\left(r^{2}=0.668;\;P_{uncorr}=1.13\text{ x }10^{-8}\right)$$

and for Matriarch 2:

$$WBGT_{onset}=0.8752\;x\;WBGT_{min}+4.5481\;\;\left(r^{2}=0.799;\;P_{uncorr}=2.46\text{ x }10^{-12}\right)$$

The time of sunrise was 06:32 at the start of the 35-day recording period, and was 06:42 at the end of the recording period. Sleep offset, or the time of awakening, was defined as the end of the last consolidated sleep bout, greater than 10 minutes, that occurred prior to sunrise. The time of awakening varied during the recording period, but for Matriarch 1 the average time of awakening was 05:07 (s.d. = 68 min; range = 02:05 to 06:20), for Matriarch 2 the average time of awakening was 04:56 (s.d. = 44 min; range = 03:35 to 06:20), while combined the average time of awakening was 05:01 (s.d. = 57 min) (Fig 4E). Matriarch 1 woke up on average of 87.3 min (s.d. = 66.6 min; range = 12–270 min) prior to sunrise, Matriarch 2 woke up on average of 103.9 min (s.d. = 47.7 min; range = 19–197 min) prior to sunrise, while combined the average time of awakening prior to sunrise was 95.7 min (s.d. = 57.9 min). Thus, the two elephants studied always woke up prior to sunrise and not a single substantive episode for either of the two elephants sleeping for several hours after sunrise was observed (Fig 2). In addition, the two elephants, on average, woke up 72.7 min prior to civil dawn (which occurred 23 min prior to sunrise), 46.7 min prior to nautical dawn (which occurred 49 min prior to sunrise) and 21.7 min prior to astronomical dawn (which occurred 74 min prior to sunrise) (Fig 4E). No clear correlations indicated that dawn, of any sort, played a significant role in awakening.

Due to the lack of correlation between awakening and sunrise times we examined the relationship of minimum WBGT (see above) to the timing of awakening. Awakening for Matriarch 1 occurred prior to minimum WBGT for 23 out of 32 awakening events (71.9%), while for Matriarch 2 awakening occurred prior to minimum WBGT for 24 out of 33 awakening events (72.7%) (see below for days without sleep). Matriarch 1, on average, woke up 40.45 min (s.d. = 101.8 min; range = 275 min prior to 195 min after) prior to minimum WBGT, while Matriarch 2 woke up, on average, 53.94 min (s.d. = 102.6 min; range = 205 min prior to 255 min after) prior to minimum WBGT. Thus, the two elephants studied generally woke up prior to the time of minimum WBGT (Matriarch 1: p = 0.032; Matriarch 2: p = 0.005; paired T-test), and while this is a statistically significant result, the variance is high (Fig 4E). The WBGT at the time of awakening, either before or after minimum WBGT, was, on average, 0.82 degrees higher than the minimum WBGT for Matriarch 1 (s.d. = 0.74 degrees; range = 0 to 2.99 degrees), while for Matriarch 2 it was on average 0.96 degrees (s.d. = 0.76 degrees; range = 0.03 to 3.03 degrees) higher than the minimum WBGT. Statistically significant linear correlations existed between minimum WBGT (*WBGT*_*min*_) and WGBT at awakening (*WBGT*_*wake*_) for both elephants (Fig 4D), irrespective of arousal occurring prior to or after the time of minimum WBGT, such that for Matriarch 1:

$$WBGT_{wake}=1.0218\;x\;WGBT_{min}+0.4976\;\;\left(r^{2}=0.944;\;P_{uncorr}=2.49\text{ x }10^{-20}\right)$$

and for Matriarch 2:

$$WBGT_{wake}=1.028\;x\;WBGT_{min}+0.5548\;\;\left(r^{2}=0.946;\;P_{uncorr}=3.69\text{ x }10^{-21}\right)$$

### Days without sleep

One of the more unexpected findings of the current study was the observation that on 4 specific days during the recording period the elephants did not appear to sleep. On the 17^th^ of April neither elephant showed any inactivity of the trunk that could be scored as sleep. On the 5^th^ of May Matriarch 2 showed no signs of sleep, on the 8^th^ of May, Matriarch 1 had two 5 min episodes of trunk inactivity close to sunset, and on the 14^th^ of May Matriarch 1 exhibited one 5 min episode of trunk inactivity at around 3 am. The times between main sleep episodes were: 45 h 19 min on the 16–18^th^ of April for Matriarch 1; 48 h 41 min on the 16–18^th^ of April for Matriarch 2; 46 h 00 min on the 4–6^th^ of May for Matriarch 2; 45 h 40 min on the 7–9^th^ May for Matriarch 1 (if the two short episodes are ignored); and 46 h 05 min on the 13–15^th^ of May for Matriarch 1 (if the one short episode is ignored). No specific extreme weather conditions were noted on any of these four days, nor were any correlations observed to the lunar cycle (i.e. full moon) that coincided with these days without sleep.

**Fig 5:**
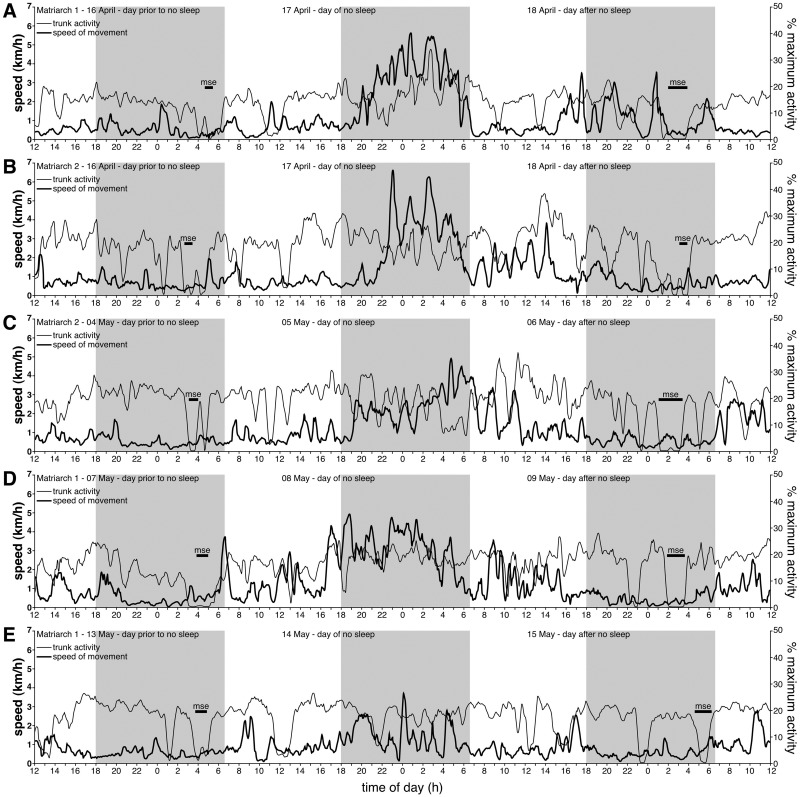
Days without sleep. Line plots of trunk activity (calculated as percent of maximum activity to allow direct comparison between animals, **thinner line**) and speed of whole body movement (calculated as distance between subsequent GPS locations divided by time in kilometres per hour, **km/h**, **thicker line**) over the day prior to the day with no sleep, the day of no sleep and the day following no sleep, for the five occasions when this occurred during the recording period. The three grey regions in each plot represent the three periods between sunset and sunrise incorporated into these plots. The plots provided are of 15 minute moving averages, which highlight the periods (and reduce the noise of the raw data) of increased and sustained bodily and trunk activity when the elephants would have been predicted to be sleeping (see Figs 2 and 3). (**A**) Matriarch 1 16–18 April, (**B**) Matriarch 2 16–18 April, (**C**) Matriarch 2 04–06 May, (**D**) Matriarch 1 07–09 May, and (**E**) Matriarch 1 13–15 May. **mse**–main sleep episode, with the horizontal bar length representing the duration of the episode.

While the activity of the elephants appeared to be following a typical pattern, at approximately 20:00 on the evening of the 17^th^ of April, both elephants appeared to become unusually active, especially in terms of speed of whole body movement as judged from GPS location, for extended periods (Fig 5). At this time the elephants were located 3.09 km apart. For Matriarch 1, the period of increased whole body motor activity appeared to last for at least 11 hours after the initiation of this increased activity, and while the activity levels dropped to normal during the daylight of the 18^th^ of April, whole body movement was still high in the afternoon and into the night until around 23:30 (Fig 5A). During the approximately 11 hours of increased activity, Matriarch 1 traversed a total distance of 32.22 km (27.01 km straight line distance). For Matriarch 2, this period of increased motor activity was maintained for at least 10 hours, but there was a sustained higher than normal activity lasting through to 21:00 on the evening of the 18^th^ of April (Fig 5B). During the approximately 10 hours of increased activity, Matriarch 2 traversed a total distance of 24.57 km (21.79 km straight line distance). On the evening of the 5^th^ of May, at approximately 19:00, the whole body motor activity of Matriarch 2 was observed to increase, though not as dramatically as on the 17^th^ of April, and this was sustained until 11:30 the following morning (Fig 5C). During the approximately 16.5 hours of increased activity, Matriarch 2 traversed a total distance of 38.09 km (28.19 km straight line distance). In the late afternoon of the 8^th^ of May, at approximately 16:30, the whole body motor activity of Matriarch 1 was observed to increase dramatically, and this increased activity was sustained through to 05:00 the following morning, but was at higher than normal levels for most of the following day (Fig 5D). During the approximately 10.5 hours of sustained increased activity, Matriarch 1 traversed a total distance of 41.68 km (36.61 km straight line distance). On the evening of the 14^th^ of May, at approximately 19:00, the whole body motor activity of Matriarch 1 was observed to increase, though not as dramatically as on the 17^th^ of April, and this was sustained until 05:30 the following morning (Fig 5E). During the approximately 10.5 hours of increased activity, Matriarch 1 traversed a total distance of 15.00 km (5.62 km straight line distance).

In the four days following these extended periods without sleep, total sleep times for Matriarch 1 were 2.4, 3.6, 2.9, and 2.3 h (2.8 h average) for the dates 18–21 April, total sleep times for Matriarch 2 were 2.0, 1.0, 2.8, and 2.1 h (1.98 h average) for the dates 18–21 April, total sleep times for Matriarch 2 were 3.6, 1.4, 1.3, and 1.3 h (1.9 h average) for the dates 6–9 May, total sleep times for Matriarch 1 were 2.7, 1.2, 2.2 and 0.8 h (1.73 h average) for the dates 09–12 May, and total sleep times for Matriarch 1 were 1.3, 1.8, 0.4 and 0.6 h (1.03 h average) for the dates 15–18 May. Thus, in only one instance (Matriarch 1: 18–21 April) does there appear to be sleep rebound, in the form of increased TST duration, following the approximately 46 hours without sleep. It should also be noted here that recumbent sleep only occurred on one of the five days following the days without sleep (Matriarch 2 on the 6^th^ of May, a 25 minute period of recumbent sleep on the left side). Thus, after the days without sleep there was, in general, no sign of additional recumbent sleep that might be interpreted as sleep rebound.

### Variability of sleep locations

**Fig 6:**
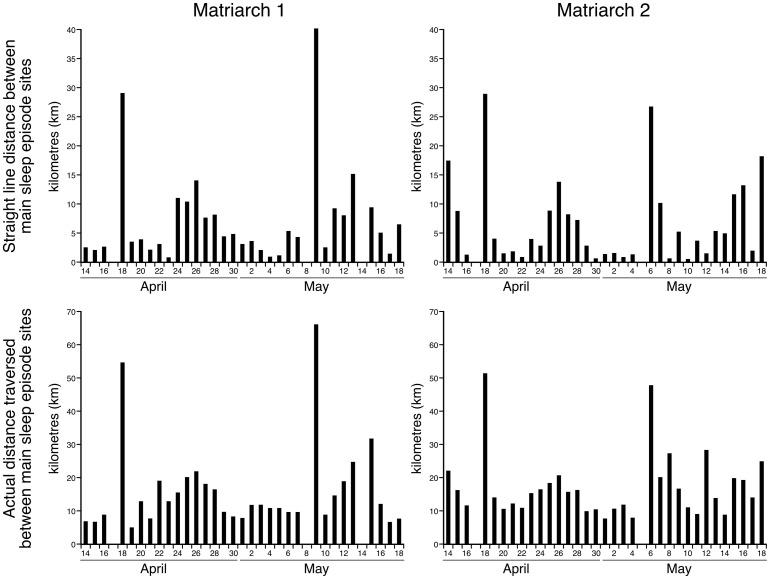
Distance between sleep sites. Bar graphs showing the straight-line distance (upper graphs) and the actual distance traversed (lower graphs) for Matriarch 1 (left graphs) and Matriarch 2 (right graphs) on each day between the sites where the major sleep episode occurred. The straight-line distance between the locations of the main sleep episodes for Matriarch 1 averaged 6.8 km (s.d. = 7.3 km; range = 0.8–34.3 km) and for Matriarch 2 averaged 6.9 km (s.d = 7.4 km; range = 0.5–29.1 km). The actual distance travelled between each sleep location between main sleep episodes averaged 15.1 km (s.d. = 10.8 km; range = 5.1–54.9 km) for Matriarch 1 and 17.3 km (s.d. = 9.9 km; range = 7.7–51.5 km range) for Matriarch 2.

**Fig 7:**
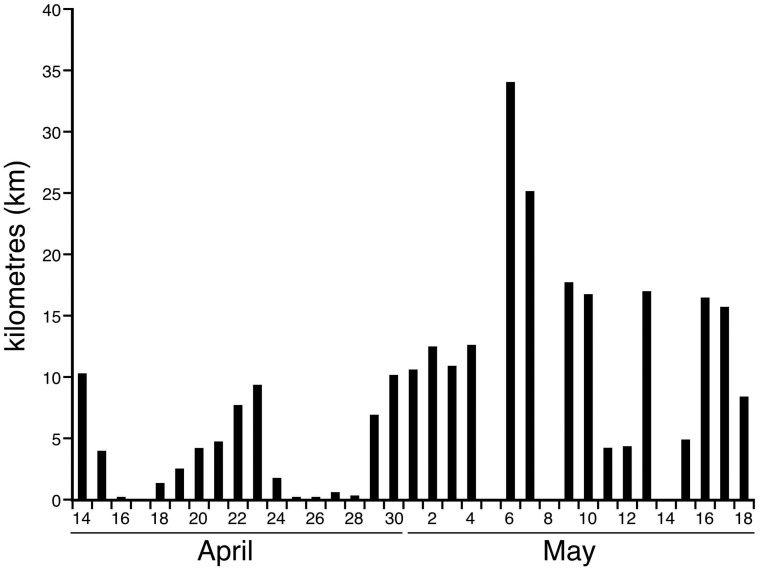
Distance between sleeping herds. Bar graph showing the distance (kilometres, **km**) between sites of the major sleep episode of Matriarch 1 and Matriarch 2 on each day of the recording period when sleep occurred. The average distance between the sites of the major sleep episode for the two elephants on the same day was 9.3 km (s.d. = 8.1 km, range = 0.15–34.05 km). The closest the two matriarchs (and their respective herds) came to sleeping in the same location was 150 m, but on other days they could be over 34 km apart.

The two elephants monitored slept in new locations on every day of the recording period (Table 3). The straight-line distance between the locations of the main sleep episodes for Matriarch 1 averaged 7.2 km (s.d. = 8.2 km; range = 0.8–40.2 km) and for Matriarch 2 averaged 6.9 km (s.d = 7.4 km; range = 0.5–29.1 km) (Fig 6). The actual distance travelled between each sleep location between main sleep episodes averaged 16.1 km (s.d. = 13.2 km; range = 5.1–66.4 km) for Matriarch 1 and 17.3 km (s.d. = 9.9 km; range = 7.7–51.5 km range) for Matriarch 2 (Fig 6). While inhabiting the same general area of the Chobe National Park, the two elephants did not sleep in the same location on any of the days recorded, meaning that the herds led by these two matriarchs did not appear to merge during the recording period, at least for sleeping. The average distance between the sites of the major sleep episode for the two animals on the same day was 8.9 km (s.d. = 7.9 km, range = 0.15–34.05 km) (Fig 7). Thus, the closest the two matriarchs (and their respective herds) came to sleeping in the same location was 150 m, but on other days they could be over 34 km apart.

**Table 3:** Co-ordinates (Latitude° S, Longitude° E) for location where the main sleep episode occurred on all days that the two elephants exhibited a main sleep episode. **X**–days without data as the elephants did not sleep on these days.

Recording day	Date	Matriarch 1		Matriarch 2	
		Latitude° S	Longitude° E	Latitude° S	Longitude° E
1	14 Apr	18.05649	25.03646	18.00428	25.01540
2	15 Apr	18.06846	25.03664	18.04721	25.04005
3	16 Apr	18.05369	25.03899	18.05442	25.03827
4	17 Apr	X	X	X	X
5	18 Apr	17.52314	24.54497	17.52053	24.55184
6	19 Apr	17.52406	24.56569	17.51347	24.57459
7	20 Apr	17.50359	24.56086	17.51045	24.58320
8	21 Apr	17.51566	24.56018	17.52118	24.58638
9	22 Apr	17.52421	24.54438	17.52635	24.58795
10	23 Apr	17.52763	24.54746	17.54774	24.59577
11	24 Apr	17.56850	24.59397	17.56258	25.00169
12	25 Apr	17.51758	25.02057	17.51683	25.02055
13	26 Apr	17.58648	25.05270	17.58578	25.05356
14	27 Apr	18.02817	25.05320	18.03105	25.05504
15	28 Apr	18.06645	25.07769	18.06569	25.07647
16	29 Apr	18.08666	25.09166	18.06577	25.05941
17	30 Apr	18.07551	25.11683	18.06859	25.06001
18	1 May	18.09006	25.10678	18.06877	25.05097
19	2 May	18.08346	25.12678	18.06336	25.05894
20	3 May	18.09076	25.11720	18.06862	25.06003
21	4 May	18.09602	25.11715	18.06757	25.05198
22	5 May	18.09865	25.11074	X	X
23	6 May	18.08029	25.13477	17.52323	25.03355
24	7 May	18.09634	25.11622	17.56784	25.06875
25	8 May	X	X	17.56390	25.06860
26	9 May	17.51938	25.01550	17.58743	25.08636
27	10 May	17.51466	25.02951	17.58523	25.08924
28	11 May	17.55977	25.05397	17.56878	25.07593
29	12 May	18.00076	25.07103	17.57795	25.07687
30	13 May	17.52121	25.04718	17.59795	25.09988
31	14 May	X	X	17.58056	25.07736
32	15 May	17.54283	25.03360	17.52180	25.05044
33	16 May	17.51535	25.02855	17.58675	25.08393
34	17 May	17.52100	25.03476	17.59588	25.07721
35	18 May	17.55674	25.02917	17.51152	25.02152

As each animal had a GPS collar attached, we could, *post-hoc*, determine the vegetation type selected by each elephant as a sleeping location by matching these sites to vegetation maps of the Chobe National Park. Both elephants showed no specific preference for the vegetation types associated with the selection of sleep sites for the main sleep episode, or the shorter sleep episodes. Matriarch 1 spent the first three days of the recording period having the main sleep episode in open to dense savanna Mopane woodland (partially lacustrine), with the remaining days of the recording period selecting sites for the main sleep episode in *Baikiaea plurijuga* (African teak) woodlands (deep sand), that on occasion was associated with dense woodland areas, or more open savanna areas, and sometimes associated with *Guibourtia coleosperma* (African rosewood or large false mopane) woodland and/or *Burkea africana* (wild syringa) dense to open savanna (reworked sands). Matriarch 2 showed a very similar pattern of vegetative selection for the sleeping site of the main sleep episode, but spent the first night of the recording period in *Baikiaea plurijuga* (African teak) woodlands, the next two nights in open to dense savanna Mopane woodland, before following a similar variety of vegetation types as Matriarch 1 for the remainder of the recording period. These observations indicate that specific vegetation types were not preferentially selected as sleeping sites by the elephants.

### Daily distance travelled, trunk activity, total sleep time and duration of the main sleep episode

**Fig 8:**
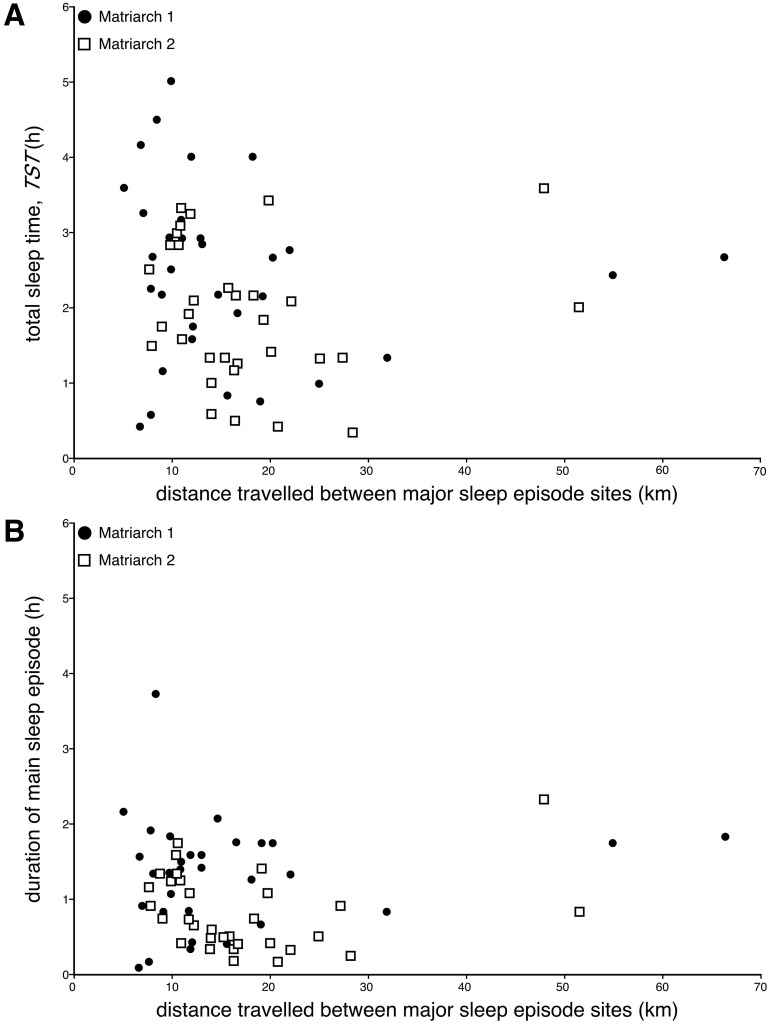
Sleep times and distance travelled. Scatterplots showing the lack of a relationship between distance travelled between the sites of the main sleep episodes (kilometres, **km**) and total sleep time (**TST**, hours, **h**) (**A**) or duration of the main sleep episode (hours, **h**) (**B**) for both Matriarch 1 (**closed circles**) and Matriarch 2 (**open squares**). While there is a trend for shorter sleep times with increased distance travelled, these trends are not statistically significant even when the days without sleep are removed from the analysis. These plots demonstrate that prior activity does not appear to affect sleep times.

**Fig 9:**
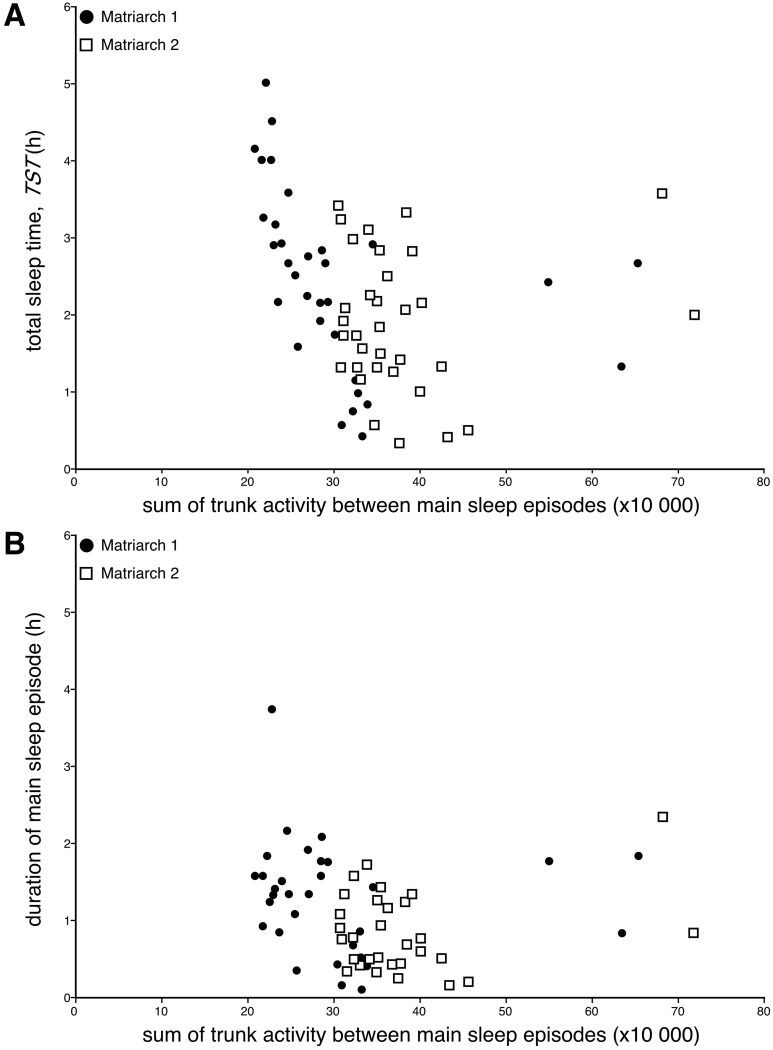
Sleep times and trunk activity. Scatterplots showing the lack of a relationship between total trunk activity between main sleep episodes and total sleep time (**TST**, hours, **h**) (**A**) or duration of the main sleep episode (hours, **h**) (**B**) for both Matriarch 1 (**closed circles**) and Matriarch 2 (**open squares**). While there is a trend for shorter sleep times with increased trunk activity, these trends are not statistically significant even when the days without sleep are removed from the analysis. These plots demonstrate that prior activity does not appear to affect sleep times.

In order to assess whether the level of activity prior to sleep influenced the duration of sleep, we examined the distance travelled between main sleep episodes and the counts of trunks movements between main sleep episodes, with the subsequent length of the main sleep episode and the associated total daily sleep time. No correlation between the distance traversed between main sleep episodes and the total sleep time or the duration of the subsequent major sleep episode was observed (Fig 8). For Matriarch 1, the average distance traversed between major sleep episodes amounted to 16.1 km (s.d. = 13.2 km; range = 5.1–66.4 km) (Fig 9A), while for Matriarch 2 the average distance traversed was 17.3 km (s.d. = 9.9 km; range = 7.7–51.5 km) (Fig 8B). In both elephants a weak trend towards having a longer total sleep time or main sleep episode with a shorter distance traversed between main sleep episodes was apparent, but these trends were not statistically significant for either individual elephant or when the data from both elephants was combined. This lack of correlation was maintained when the days without sleep, leading to a greater distance and time between major sleep episodes, were excluded from the analysis.

Similarly, no correlation between the actual amount of trunk activity (sum of all counts recorded by the actiwatch) between main sleep episodes and the total sleep time or the duration of the subsequent major sleep episode was observed (Fig 9). For Matriarch 1, the average sum of counts of trunk activity between major sleep episodes amounted to 302 494 (s.d. = 109 721; range = 208 847–653 189) (Fig 9A), while for Matriarch 2 the average sum of counts of trunk activity between major sleep episodes amounted to 376 255 (s.d. = 91 771; range = 306 012–718 812) (Fig 9B). In both elephants a weak trend towards having a longer total sleep time or main sleep episode with a smaller sum of trunk activity between main sleep episodes was apparent, but these trends were not statistically significant for either individual elephant or when the data from both elephants was combined. This lack of correlation was maintained when the days without sleep, leading to a greater distance and time between major sleep episodes, were excluded from the analysis.

## Discussion

The present study of two wild matriarch African elephants has revealed a number of important observations regarding sleep in their natural habitat, that are both similar to, and that differ significantly from, previous observational studies made on captive elephants (Table 1). Briefly, the elephants studied were primarily nocturnal, polyphasic sleepers that have an average of 2 hours of sleep per night. We observed that the elephants sleep in either a standing or recumbent posture, although neither sleep type occurred on all nights of the recording period, with recumbent sleep occurring roughly every third or fourth night, and for 5 out of 70 nights of recording no sleep was observed. Sleep onset and offset showed distinct relationships to the environmental conditions, the elephants did not use the same sleep site during the recording period, and total sleep time was not related to the amount of activity between sleep periods. The current observations provide a far richer story of elephant sleep in their natural environment than would have been suspected from studies in zoo or circus animals and underscores the need to study sleep more broadly in the natural habitat in addition to controlled settings.

### Limitations of the current study

While we feel the current study has produced a number of exciting and interesting results regarding sleep in wild elephants, there are three specific aspects of the study that need to be highlighted in order to properly contextualize the data presented here. First, the study was undertaken on only two adult female elephants that were both matriarchs of their respective herds. In this sense, the sleep patterns and times outlined herein might not be representative of wild elephants as a whole, as no males were included in the study, no non-matriarch females were included, and the responsibilities of the matriarch of a herd may affect sleep patterns. Second, Matriarch 2 was nursing a calf of less than 1 year old which appears to have led to a shorter overall average amount of sleep, an increased fracturing of sleep (Fig 2) and less recumbent sleep (Fig 3) in this animal compared to the non-nursing Matriarch 1. However, it should be noted that elephant calves nurse until they reach approximately 3–5 years of age, or until another sibling is born which can occur 3–4 years after the first calf, thus it would be more rare for a matriarch elephant to be without a suckling calf than to have a suckling calf [15]. Despite the differences between the nursing and non-nursing elephants, the remaining parameters measured appeared to be very similar between the two animals, indicating that we can, at the very least, make generalizations about sleep of wild, free-roaming matriarch elephants from the current study.

The third aspect of the current study that needs to be highlighted is that we did not use polysomnography (PSG) to record true physiological sleep; rather, we used actigraphy of the trunk as a proxy to infer sleep. As mentioned earlier, we are aware that actigraphy can overestimate sleep times, due to the difficulty in determining quiet wake from sleep using actigraphy and that trunk actigraphy has never been validated against PSG in the elephant, but as observed in captive studies, inactivity of the trunk is a clear indication of behavioural sleep in the elephant [1, 8]. Moreover, if actigraphy in the elephant does overestimate sleep times, then the results provided herein should be considered maximum sleep times for wild African matriarch elephants. Unfortunately, due to the anatomy of the elephant skull, with a large, extensively trabeculated frontal sinus overlying the brain, that has a depth of up to 30 cm in adults [21,22], the surgery required to implant EEG and other recording electrodes, especially in wild elephants, would be highly invasive, both ethically and technically problematic, and may interfere with other aspects of the elephant’s behaviour that would affect sleep. Thus, we feel the approach taken herein to be the least invasive approach we can currently undertake in order to study sleep in free-roaming wild elephants. Given the results of the current study this approach does appear to be a very useful way to undertake this sort of study that limits any ill-effects on the subjects of study while still providing useful and interesting results that are likely an accurate reflection of sleep in wild elephants. It should be noted here that all implants and collars were removed from the two elephants at the end of the 35-day recording period without detriment to the elephants.

### Comparisons to studies of elephant sleep in the captive setting

Our observations of sleep in the wild free-roaming matriarch elephants show features both in common with previous studies of elephant sleep in captivity (Table 1) and significant differences. Similar to observations made in the captive setting, the wild elephants slept mostly at night (between midnight and dawn), showed polyphasic sleep, standing sleep, recumbent sleep, and similar recumbent sleep episode lengths. However, in contrast to the studies in the captive setting, the wild elephants: (1) on average slept substantially less each day; (2) showed several days without any form of identifiable sleep; (3) showed far less recumbent sleep; (4) recumbent sleep did not occur daily; (5) sleep onset and offset times appeared to be related to environmental conditions; (6) appeared to select novel sleeping sites each night (Table 3); and (7) did not show any form of sleep “rebound” in response to disturbances.

Our study has demonstrated that the two elephants we recorded appear to sleep somewhere between 1 to 4 hours less than previous reports of total sleep time in captive elephants [2,3,8], and approximately 1.2–1.3 hours less than previous studies in the wild [5]. This finding is perhaps not surprising in the light of similar reductions in sleep times being observed in wild sloths compared to captive sloths [13], and the different methodology used when establishing activity and rest in the elephants in the wild, from focal observations over several months and seasons [5], to the continuous remote monitoring undertaken in the current study over a distinct period of clement weather. In addition, we noted several days without sleep (see below for Discussion), that has only been mentioned once in previous studies of elephant sleep in captivity, and this when the elephants, which were part of a circus, were being moved from one site to another [1]. Recumbent sleep occupied 12–15% of total sleep time in the two wild elephant recorded herein, being between 13–21 minutes per day. Interestingly, recumbent sleep only occurred every third to fourth day, although it has been noted that occasional days without recumbent sleep do occur in captive elephants [12]. The amount of recumbent sleep in the wild elephants is substantially less than the 2.6 h/day [12] and 4.1 h/day [8] reported for recumbent sleep time in the captive setting; however, in agreement with Tobler [8] we found no bias for recumbent sleep to occur on the right or left side. Despite the fact that recumbent sleep only occurred sporadically in the wild elephants, when an episode of recumbent sleep did occur, it had an average length of 38–65 min, which is not greatly dissimilar to the 72 min (African and Asian), 39 min (African) and 66 min (Asian) average recumbent sleep episode lengths reported in the captive setting [8,12]. Thus, when recumbent sleep did occur in the wild elephants, the length of a recumbent sleep episode did not appear to differ markedly from that observed in the captive setting. Unfortunately, the captive setting cannot provide data about the natural environmental conditions, as they differ substantially from the normal habitat of the African elephant, and the captive setting does not allow for the selection of a novel sleep site each night. Thus, there are both interesting similarities (standing and recumbent sleep and similar recumbent sleep episode lengths) and differences (reduced standing and recumbent sleep times, sleep not occurring each day, recumbent sleep occurring every few days, environmental conditions predicting sleep onset and offset times, novel daily sleep sites, and lack of rebound when sleep deprived) when comparing sleep in wild and captive elephants.

These lists of similarities and differences highlight two important points in the field of comparative sleep studies. First, studies of “exotic” species in the captive setting, such as elephants, can reveal basic features of sleep in the species studied that can be translated to studies of sleep of the same species in their natural habitat. As such, captive studies provide information that is of importance in understanding the results of studies of sleep in the natural habitat and what features should be measured and examined carefully (such as standing and recumbent sleep in the elephant). Second, it is important to consider that the captive setting likely will not reveal the full suite of potential behaviours and factors associated with and affecting sleep in the natural habitat. This in turn makes quantitative data of sleep studies in the captive setting unreliable for the formulation of theories and correlates of sleep in an evolutionary context (e.g. [13]). Thus, sleep studies in captivity and the natural habitat can work in concert in order to arrive at data that is truly reflective of the various aspects of sleep in a particular species of interest. While this is not always readily achievable, especially for smaller species, it appears that currently this bilateral approach is achievable for larger mammals (e.g. [23]).

### Are elephants really the shortest sleeping mammals?

At 2 h per day, the total sleep times recording for the two matriarch elephants in this study are substantially less than that recorded for sleep times in captive elephants and from observational studies of wild elephants (Table 1). The remote monitoring techniques employed in the current study ensured that the elephants recorded from were undisturbed by the experimenters, and indeed, the actigraphy technique tends to overestimate the amount of sleep, meaning that the actual true physiological sleep times in wild elephants might be even lower than reported here. The average total daily sleep time of 2.0 h for these two elephants is less than that reported for any previously studied mammal [24–28], even the significantly larger bodied gray whale that appears to sleep for at least 9 h per day [29], and the giraffe, another large bodied African mammal, which, in captivity, is reported to sleep for around 4.6 h per day [25]. The next shortest sleepers of mammals, after the elephants, appear to be the domestic horse, which sleeps for 2 h 53 min per day [30] and the domestic pony, which sleeps for 3 h 20 min per day [31]. Thus, at present, it appears reasonable to conclude that the elephant is, as far as we know, the shortest sleeping mammal.

The regressions of body mass against total sleep time presented earlier in this manuscript appear to provide perhaps the most direct explanation of the 2 h total sleep time for elephants. While 2 h per day does fall well below the regressions created (Fig 1), it must be noted that these regressions are based mostly on data obtained from captive animals and that in their natural habitat these animals likely sleep far less than in captivity (e.g. [13]). Thus, if we had enough data of total sleep time from mammals in their natural habitat, it would appear that total sleep time in the elephant might be reliably predicted from such a regression.

### REM sleep is likely to be unusual in the elephant

The question of REM, or paradoxical, sleep in the elephant remains open, mainly due to the difficulties in determining REM sleep without polysomnographic recording, which as mentioned above would be extremely difficult to undertake in elephants; however, our findings do have some bearing on this issue. The only previous report of REM sleep behaviours in sleeping elephants was provided by Tobler [8], who indicated that signs of REM sleep, such as twitches and jerks of the ears, leg, tail and trunk, as well as irregular breathing and occasional loud vocalizations, were observed only during recumbent sleep; no signs of REM sleep were reported for standing sleep. In mammals more generally, one of the principal polysomnographic signs of REM sleep is muscle atonia [14]; thus, it would be reasonable to *a priori* presume that REM sleep in the elephant likely only occurs when it is in recumbency, as it would require muscle tone to maintain the standing sleep position. The domestic equids have been reported to enter REM sleep when in lateral recumbency and not in standing sleep [30,31], but a later report indicates the possibility of brief episodes of REM sleep while standing as well as longer REM sleep episodes during sternal and lateral recumbency in the horse [32].

If it is true that the elephant only experiences REM sleep when recumbent, then the findings of the current study indicate that the elephant will only enter REM sleep on every third or fourth day on average. As described above, both wild elephants only experienced recumbent sleep for 10 out of the 35 days of recording, limiting their potential opportunities to enter REM sleep if this only occurs during recumbency. This would then indicate a very different way of obtaining the necessary amount of REM sleep in the elephant compared to that seen in other mammals studied to date, with REM sleep not occurring each day. Given the capacity for the storage and recall of memories by the elephant [33], this potentially intermittent form of REM sleep, where elephants can exhibit no potential REM sleep for up to 9 days (Fig 3), may pose additional problems for functional interpretations of REM sleep related to memory consolidation [34,35]. For the elephant, this would mean that the absolute maximum percent of TST that is occupied by REM sleep would be 12–15%, which appears high compared to its close relatives the manatee (1%) [36] and the rock hyrax (0.4%) [37], but less than that seen in domestic equids which show REM sleep in lateral recumbency (~24%) [31]. In addition, the fact that when recumbent sleep did occur in the wild elephant the episode lengths are similar to those reported in the captive setting (see above), adds additional support to the concept that REM sleep only occurs in recumbency in the elephant. An absence of REM sleep for up to 4 days has also been reported for the walrus [28].

As an alternative explanation to REM sleep only occurring every 3 to 4 days in the elephant during recumbent sleep, standing sleep in the elephant may show some resemblance to sleep seen in birds, with short sleep episodes showing roughly 90% slow wave sleep and 10% REM sleep, often while standing [38], or in the domestic horse, with brief episodes of REM sleep (16–23 s) occurring during standing prior to arousal [32]. In this possible scenario, REM sleep could occur on an almost daily basis in the elephants while in standing sleep (except for those days with no sleep at all), but may occupy a small percentage of total sleep time (similar to that seen in the closely related manatees and hyraxes). This possibility is supported by the existence of a unique medially located subdivision of the locus coeruleus complex [16], which, hypothetically, could continue to depolarize during the potential standing REM sleep episodes (while the firing patterns of the remainder of the typically mammalian locus coeruleus neurons follow a standard mammalian pattern and do not depolarize during REM sleep episodes), and thereby maintain just enough muscle tone for the elephant to have short episodes of REM sleep without needing to be in recumbency. During these hypothetical short episodes of standing REM sleep the elephants may be leaning against trees or rocks for support and balance, as has been noted in observational studies of elephant sleep in the wild and captive settings [4,8], and this hypothetical standing REM sleep may coincide with the periods of “bobbing”, “weaving” or “swaying” movements of the head noted during standing sleep [4,8] similar to the neck lowering and leg buckling seen in the domestic horse [32]. In this scenario, recumbent sleep may represent periods where sleep intensity is much higher than during standing sleep. Unfortunately, the true nature of REM sleep in the elephant will only be determined once the technically and ethically difficult polysomnographic recordings have been made, but whatever the outcome, it appears clear than REM sleep in the elephant will be unusual in comparison to that seen in many other mammals.

### Days without sleep

One of the more remarkable series of observations made in the current study were the days without sleep observed in the two elephants studied. A total of 5 out of 70 days of recording (3 days for Matriarch 1, and 2 days for Matriarch 2) showed no clear signs of sleep. These days appeared to be associated with major disturbances to the elephants. The elephants showed no signs of inactivity/sleep for periods averaging 46 h 21 min, with clear signs of the animals being agitated for 10 to 16.5 h (based on increased speed of whole body movement and higher trunk activity levels). During these times the elephants traversed an average distance of 30.31 km from the beginning of the disturbance through to the time when activity and body speed levels returned to close to normal, thereby putting substantial distance between themselves and the location of the initial disturbance. Similar periods without sleep have been observed in the walrus [28].

There are three equally possible circumstances that may have caused these nights without sleep. In the Chobe National Park it has been well documented that particular large lion prides predate upon elephants [39,40]. While these lions most often predate upon male elephants aged between 4–15 years [39,40], these elephants are still associated with a herd led by a matriarch. Therefore, it would be of importance for the matriarch to lead her herd a long way from danger. Thus, predation pressures from lions may be one root cause of the nights without sleep. A second possibility is poaching of elephants by humans [41], which would likely cause a similar lack of sleep and major movement of the elephant herds led by the matriarchs from which we recorded. A third possibility is that the herds may have been disturbed and subsequently harassed by a bull elephant in musth. As the elephant herds may contain oestrus females they would be attractive to a bull in musth, and the persistence of the bull elephant over time in his attempts to mate would also explain the large distance traversed by the matriarch and her herd during these nights without sleep. Whatever the root cause of these disturbances, it is clear that wild female elephants are likely to experience roughly 7% of their nights without any signs of sleep. Further to this, there appears to be no specific sleep “rebound”, in terms of an increased total sleep time, or increased amounts of recumbent sleep, in the days following these nights without sleep and sustained increased activity. Thus, the loss of a night’s sleep does not appear to have a detectable or distinct effect on elephant sleep as far as can be determined from what we have recorded. Despite this, it is possible that following these nights without sleep, the potential sleep deficit might be compensated for by increased sleep intensity, i.e. deeper sleep rather than longer sleep. This might be tested in future by placing collars on the elephants that allow for remote activation of a stimulus (such as a vibrating collar) that would awaken the animals and thus provide arousal thresholds between different nights of sleep, and even different types of sleep (standing *vs* recumbent) in the elephant.

### Environmental conditions and sleep

Having recorded sleep in the natural habitat of the elephant we were very interested in whether any of the weather and lunar parameters measured showed specific relationships to the sleep data accrued during the study. For the most part, sleep durations, onsets, offsets and sleep types appeared to occur independent of individual weather and lunar parameters, but both ambient air temperature and relative humidity appeared to be related to sleep onset and offset times. These relationships became far stronger when a multifactorial measure, the wet-bulb globe temperature (WBGT, calculated using ambient air temperature, relative humidity, wind speed and solar radiation) that provides an estimate of the perceptual gestalt of environmental conditions, was used. In fact, WBGT explains between 67 to 80% of the variance in sleep onset times, while WBGT explains approximately 94.5% of the variance in sleep offset, or awakening, times. These results clearly indicate that sunrise and sunset times are not the cues driving the onset and offset of sleep in the elephant, and that this gestalt measurement of perceived environmental conditions is a far more accurate predictor of when elephants will enter or awake from sleep. This observation would be strengthened by the study of additional elephants, especially large males, and during different seasons, but the strength of the explanatory value of this perceived environmental condition is clearly of interest not only for elephants, but for other species sleeping in their natural habitats. Recent observations of San people living a traditional, pre-industrial, lifestyle also indicate that solar light levels do not influence the timing of sleep onset or awakening, rather ambient air temperature better explains sleep onset and offset times [42]. Thus, it appears that sleep onset and offset times in large diurnal mammals are best explained by factors such as ambient air temperature and relative humidity, or a gestalt measure of perceived environmental conditions, rather than sunrise and sunset. Further investigations across different species will test the correlations observed in elephants and humans living a traditional lifestyle and see how generally applicable these correlations prove to be. These correlations do not exclude entrainment of general circadian rhythms by light in large mammals in their natural environment, but rather explain the timing of sleep in the daily cycle of these species.

**Fig 10:**
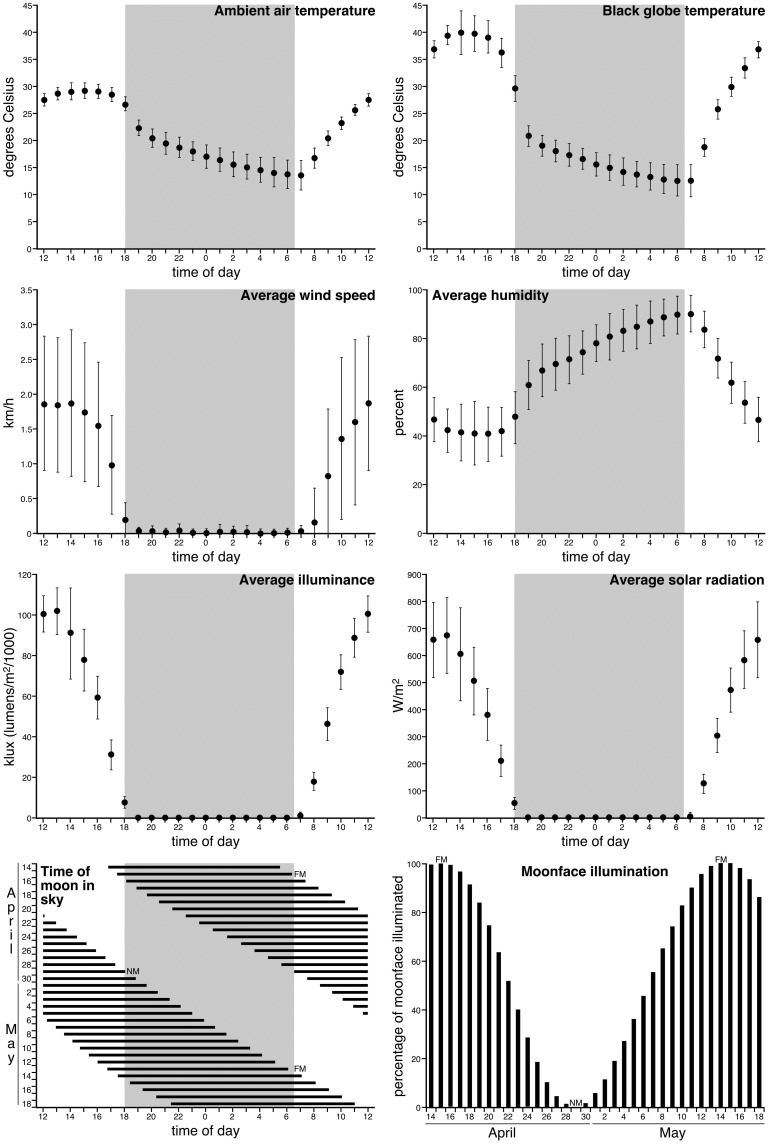
Weather parameters during the recording period. During the recording period no extreme weather conditions were encountered. The upper six graphs in this figure show the hourly averages (with the bars representing 1 standard deviation) for the 35-day recording period. The grey regions represent the period between sunset and sunrise. **km/h**–kilometres per hour; **m**^**2**^ –metres squared; **W/m**^**2**^ –watts per metre squared. The lower two graphs represent the time the moon was in the sky (**black horizontal lines**) in relation to sunset and sunrise and the percentage of the face of the moon that was illuminated by the sun. **FM**–full moon; **NM**–new moon.

A potential relationship between recumbent sleep and the days (±7 days) around the new moon is apparent for the two elephants recorded from (compare Figs 3 and 10). We observed that 70% of recumbent sleep time (15 out of 26 episodes) occurred when the face of the moon was less than 50% illuminated, but this relationship was not statistically significant. Such a relationship would of course be of interest, as it would indicate that recumbent sleep would preferentially occur in the elephant during the periods when lunar illumination is as it lowest, thereby making the elephants less detectable to predators allowing them to enter a form of sleep where the risk of predation would be higher, as it would take time to stand up and escape potential attack. Similarly, weak trends to less total sleep time around the full moon and more sleep around the new moon were noted. However, these potential relationships remain speculative and require further investigation to determine if these potential relationships are indeed real. Despite this, it appears that environmental conditions do play a major role in shaping sleep in the wild free-roaming elephant and that lunar phases may also have an effect.

### Multiple factors likely shape elephant sleep architecture

Comparative sleep studies have identified a variety of factors, with associated hypotheses, that have been linked to the shaping of the adult phenotype and architecture of sleep across vertebrates, especially so in mammals [14,26,43–50]. Of these factors that have been identified previously, the current study reveals that at least body mass, risk of predation, environmental conditions, trophic position, and the phylogenetic history of the species are involved in shaping sleep in the elephant in its natural habitat. It is probable that all these factors play a role in the shaping of the adult phenotype of sleep in the elephant in its natural habitat, but it is also likely that these factors play a shaping role at different temporal scales, from a single day, the course of a lifetime, to generations, to that of deep evolutionary roots.

As discussed above, total sleep time in the elephant is the shortest recorded of any mammal to date, and that their large body mass appears to be the major factor leading to this overall short daily sleep time. However, in addition to body mass, the apparent risk of predation (as evidenced by days without sleep and distances travelled), and the low trophic position of elephants (and their large intake of low caloric food necessitating a large proportion of the day devoted to eating), may also play a role in shaping the low total sleep time observed. We have also observed that environmental conditions play a significant role in the timing of sleep onset and offset in the elephant, and perhaps in sleep duration, and thus environmental conditions appear to play a significant role in shaping the characteristics of elephant sleep.

The African elephant is a Eutherian mammal, and as such it is likely that sleep will be composed of both non-REM (or slow wave) and REM sleep [45]; however, it is also an Afrotherian mammal and studies of closely related species, manatees and hyraxes, have indicated that within this mammalian superorder there is substantial variance in sleep architecture and states. The fully aquatic Amazonian manatee spends 6.75 h sleeping per day, mostly in slow wave sleep (25% of which is unihemispheric slow wave sleep) and 1% of total sleep time in REM sleep [36]. The rock hyrax, while spending around 7 hours per day asleep, shows not only slow wave and REM sleep (REM being 0.4% of TST), but also a novel sleep state (3% of TST) [37], seemingly associated with variances in the pontine cholinergic system [51]. Thus, the two closest extant relatives of the elephants do show sleep that is not typically mammalian [45], which given the variances in the sleep-associated nuclei noted for the African elephant [16] may indicate that, if polysomnographic recordings of sleep in the elephant were undertaken, novel sleep states, associated with slow wave or REM (REM may form a very small portion of TST in the elephant), may be present in the elephant.

Despite these speculations, we have identified several factors that potentially contribute to the shaping of sleep architecture of the extant elephant, and these may act over different temporal scales. For example, the presumption that elephant sleep is composed of both slow wave and REM sleep, relates to their mammalian phylogenetic roots, but that an unusual form of REM may be present appears to be related to their Afrotherian ancestry. Thus, the long temporal scale of phylogenetic history of a specific species cannot be ignored when attempting to understand extant sleep architecture. In fact, it appears that the large size of the Proboscidean body evolved approximately 40 mya ago [52], indicating that the ancestors of the extant elephants may have been short sleepers for millions of years. Environmental effects (weather conditions, lunar phases), nutritional requirements, and predation risk may also shape sleep architecture in the elephant, but could potentially act at a much shorter temporal scale, from days to generations. The consideration of the temporal scale on which these factors may act to shape sleep architecture is of importance in understanding the evolutionary history and variance of sleep in extant mammals.

## Materials and methods

The University of the Witwatersrand, Animal Ethics Screening Committee approved the research reported herein (Clearance Certificate No. 2013/16/04) and the Botswana Ministry of Environment, Wildlife and Tourism, Department of Wildlife and National Parks approved the use of the elephants under their care.

### The location–Chobe National Park, Botswana

The current study was undertaken in Chobe National Park, situated in northern Botswana. Chobe National Park encompasses an area of approximately 11 700 km^2^, and has four distinct ecosystems, the Serondela (or riverfront) area, the Savuti Marsh area, the Linyati Marsh area, and between the two marsh regions, a hot and dry hinterland area made up mostly of the Nogatsaa grass woodland. While incredibly rich in terms of both animal numbers and diversity, this park also houses more than 17 000 African elephants, one of the highest concentrations of elephants in Africa [41]. Due to the size, range and healthy population of adult elephants, the Chobe National Park provided an excellent location for this study. In addition, in comparison to other large national parks, the tourist load, at around 250 000/annum is substantially lower, and night drives are not permitted in Chobe National Park, both of which will allow the elephants to be active and sleep with minimal human disturbance.

### The subjects

In the current study, two adult female African elephants, both matriarchs of their respective herds were chosen for study. **Matriarch 1** was estimated to be approximately 30 years of age (based on dentition) [53], with an estimated body mass of 3400 kg (based on a hip height regression) [54], and was considered a “left-trunker” due to the high rugosity and intense chlorophyll staining of the distal left edge of her trunk. Matriarch 1 did not appear to be pregnant, nor nursing a calf and was in good general condition (as assessed by an experienced wildlife veterinarian). **Matriarch 2** was estimated to 37 years of age, with an estimated body mass of 3000 kg, was also in good general condition, but was clearly lactating and observed to have a calf of between 6–12 months of age. Matriarch 2 was considered to be a “right-trunker” due to the high rugosity and intense chlorophyll staining of the distal right edge of her trunk.

### Immobilization, anaesthesia and surgical procedures

For the implantation and removal procedures, each animal was immobilized with an injection of Thianil and 50 mg of Azaperone (i.m. using a pressurised dart gun from a helicopter). Matriarch 1 received a single 12 ml injection of Thianil, while Matriarch 2 received an initial dose of 15 ml of Thianil, with a 1 ml supplementary Thianil dose 15 minutes after the initial dose. Each elephant received a 60 ml i.m. injection of the antibiotic Penicillin, as well as a 50 ml i.m. injection of the analgesic agent Phenylbutazone. To reverse the immobilization, each elephant received a 3 ml i.v. injection of naltrexone hydrochloride. The time each elephant spent immobilized for the actiwatch implantation and collaring procedure was 34 minutes for Matriarch 1 and 70 minutes for Matriarch 2. For the removal of the implanted actiwatch and the collar each animal received similar doses of all agents, but were only immobilized for 14 (Matriarch 1) and 19 (Matriarch 2) minutes. As both elephants were matriarchs of their herd we needed to use the siren on the helicopter to move the remainder of the herd away from the immobilized matriarchs. Upon reversal of the anaesthesia, the matriarchs would stand, vocalize, and then move to rejoin their herd. This procedure did not have any marked effect on the herd.

Once immobilized and lying in a lateral recumbent position, a region approximately 150 cm from the tip of the trunk, on the lateral aspect, was selected as the implant site. The skin over the implant site was initially washed with clean water and scrubbed with a stiff brush to remove surface dirt. The area was then scrubbed again, but with detergent to remove the remainder of the visible dirt and rinsed. The area was then scrubbed with 10% Chlorhexidine (CHX, Chlorhexidine digluconate 0,5% m/v, ethyl alcohol 75% m/v, Kyron Laboratories) and rinsed with sterile water, following which the area was sprayed with CHX and wiped with sterile cotton gauze. This procedure left the skin surface as aseptic as possible under field conditions. A sterile drape, with a 10 cm x 3 cm window was then placed over the cleaned trunk skin and tucked around the trunk to maintain the aseptic conditions. A small incision, approximately 5 cm in length, was made horizontally in the skin, and a subcutaneous pocket of approximately 15 cm in depth was made towards the tip of the trunk using sterile surgical instruments. The actiwatch, which was coated in an inert wax and had been sterilized using formalin pellets in a clean plastic container for 24 hr, was rinsed with sterile saline and then placed within the subdermal pocket. Subcutaneous stitches were made in the lower aspect of the initial incision, and superficial stitches made to draw the two sides of the incision together to seal the wound. The incision was then cleansed with CHX and sprayed with Necrospray (active ingredients Oxytetracycline hydrochloride, 40 mg, gentian violet 4 mg, Bayer Animal Health) to prevent topical infection. In both elephants the wounds healed very well and neither elephant suffered any noticeable ill effects from the implantation procedure. For removal of the actiwatch implant, exactly the same process was followed, except for the creation of a sub-dermal pocket, as the skin incision was made directly over the implanted actiwatch. Some subcutaneous scarring was noticed around the implant, but no infection was observed. The collar was also attached and removed during the implantation and removal procedures. Matriarch 1 was implanted and collared on the 9^th^ of April, 2014, with removal of the implant and collar on the 19^th^ of May, 2014. Matriarch 2 was implanted and collared on the 8^th^ of April, 2014, with removal of the implant and collar on the 19^th^ of May, 2014.

### Actiwatches

The actiwatch is a wristwatch size, ambulatory device that is commonly used for measuring sleep in humans. Within each actiwatch is a piezoaccelerometer device connected to a microchip that sums and records the number of acceleration events per minute. The *Actiwatch Spectrum* (Philips Respironics) was used in the current study. These devices have a mass of 25 g and approximate dimensions of 35 x 35 x 12 mm. Each actiwatch was calibrated and programmed (data acquisition rate set at 1 min intervals) with the Philips Respironics Actiware 5 software, prior to implantation. Following removal of the wristband the actiwatches were insulated with standard electrical insulation tape and covered in 2 coats of biologically inert wax (SasolWax 1276, Sasol, Johannesburg, South Africa).

### Collar design and function

Two standard elephant collars, manufactured by Africa Wildlife Tracking (Pretoria, South Africa), were used in the current study. Each collar had a GPS system and a 3D accelerometer that recorded data to an inbuilt logger. The GPS system recorded the precise location of the animal each minute during the entire recording period. The 3D accelerometer recorded acceleration events in the X, Y and Z planes, which were then used to determine whether the elephant was standing or in recumbency. Each collar weighed approximately 15 kg, and weights of 10 kg were used to keep the collar in the correct position for the recordings made by the 3D accelerometer. Following retrieval of the collar, the data was downloaded and stored in Microsoft Excel for analysis.

### Weather parameters during the recording period

In order to record environmental conditions we used a solar powered weather station with a data logger built by Environdata (www.environdata.com.au/easidatamark4), which recorded 8 channels of information. The station was installed at the start of the recording period, and was located no farther than 40 km from the elephants during the recording period. The weather station collected environmental data during the entire recording period (each information type being recorded once every 10 min). Over the 35 day recording period, the instrument recorded: air temperature, black globe temperature, relative humidity, wind speed, wind direction, rainfall, illuminance (lux) and solar radiation. During the recording period, no extreme weather events occurred (Fig 10). Air temperatures ranged from a minimum of 8.86°C to a maximum of 32.11°C, black globe temperatures ranged from a minimum of 7.35°C to a maximum of 46.32°C and the relative humidity ranged from 22.30% to 98.53%. Wind speeds ranged from 0–11.35 km/h, with the predominant wind direction being 152° (south-westerly), and no significant rainfall occurred during the recording period. Illuminance levels ranged from 0 kilolux (klx = lumens/m^2^/1000, at night) to 136.1 klx (during the day), with solar radiation levels ranging from 0 W/m^2^ (at night) to 939.81 W/m^2^ (during the day). The period of daylight decreased during the recording period, from 11 h 32.4 min to 10 h 52.8 min, the average daily decrease of the daylight period being 1 min 8 s. Sunrise occurred at 06:32 at the start of the recording period and 06:42 at the end of the recording period, with civil dawn occurring 23 min prior to sunrise, nautical dawn 49 min prior to sunrise and astronomical dawn 74 min prior to sunrise. Sunset occurred at 18:11 at the start of the recording period and 17:53 at the end of the recording period, with civil twilight occurring 22 min after sunset, nautical twilight 47 min after sunset and astronomical twilight 73 min after sunset. Thus, all weather parameters were well within the normal range for the region. In addition, the lunar cycle was monitored, with full moons occurring on the 15^th^ of April and 14^th^ of May, with the new moon occurring on the 29^th^ of April, during the recording period. We also noted moonrise and moonset times, percentage illumination of the moon face (Fig 10), as well as distance of the moon from the study site (Table 2).

### Analysis

Data from the 14^th^ of April through to the 18^th^ of May was included for analysis (removing the data for three to four days following implantation and collaring, allowing the elephants time to habituate to the new conditions of a collar and implant). Phillips Respironics Actiware 5 was used to retrieve the recorded data from each of the implanted actiwatches. The raw data were exported to Microsoft Excel where they were manually scored and analysed. Data obtained from the actiwatches were scored in 1 min epochs as either active or inactive. For an epoch to be scored as active the actiwatch had to have an activity score greater than zero. Inactive epochs were scored when the actiwatch recorded an activity score equal to zero. From five consecutive 1 min periods of scored data, the modal state for 5 min was determined and used to calculate total inactive/sleep time, number of inactive/sleep episodes, the average duration of inactive/sleep episodes, timing of sleep, and for all analysis of sleep in relation to other parameters measured (such as GPS location, standing *vs* recumbancy, and environmental conditions such as wet-bulb globe temperature, see below).

The collar attached to the elephants provided the data on GPS location and the speed of animal movement (calculated as distance between GPS locations divided by time). This data was used to calculate the distances travelled between specific points of interest (such as sleep location, or when the animals did not sleep). The gyroscope in the collar provided acceleration data in three planes (X, Y and Z planes) and this was used to calculate when the animal was in recumbency or standing. The resultant of the three vectors was calculated initially to determine movement related acceleration. Episodes where the resultant acceleration equalled 1 g (± 0.1 g) (indicative that the animal was not accelerating) were used in the subsequent analysis of recumbency. The acceleration in both the Z and X planes was inspected to determine if the animal was standing or lying down (+Z g value = standing; -Z g value = lying down) and laterality during recumbency (+1 g in X plane = right; -1 g in X plane = left). The periods in recumbency were then correlated with the actigraphy that indicated an inactive trunk to determine how much of the sleep time was spent in recumbency and on which side of the body the animals were lying down during recumbent sleep. GPS locations of the sites of the main sleep episodes (determined from the actigraphy) were correlated with vegetation maps of the Chobe National Park in order to determine what vegetation types the elephants selected for sleeping.

While we measured several environmental parameters, we chose to analyse elephant sleep in relation to Wet-Bulb Globe Temperature (WBGT), as this measure of apparent temperature is used to estimate the combined effect of ambient air temperature, relative humidity, solar radiation and wind speed. Thus, we could compare elephant sleep to an environmental gestalt rather than individual parameters. In order to calculate WBGT we initially calculated wet-bulb temperature from the measures of relative humidity and ambient air temperature using the equation provided by Stull [55]:

$$T_{w}=T\;x\;atan\left[0.151977\left(RH\%+8.313659\right)0.5\right]+atan\left(T+RH\%\right)–atan\left(RH\%-1.676331\right)+0.00391838\left(RH\%\right)1.5\;x\;atan\left(0.023101RH\%\right)–4.686035$$

where *T* is ambient air temperature (which we measured directly), RH% is relative humidity in percentage (which we measured directly), and *T*_*w*_ is natural wet-bulb temperature. We then calculated WBGT using the equation provided by Liljegren et al. [56]:

$$WBGT=0.7T_{w}+0.2T_{g}+0.1T$$

where *T*_*w*_ is natural wet-bulb temperature (calculated as shown above), *T*_*g*_ is black globe temperature (which we measured directly), and *T* is ambient air temperature (which we measured directly).
